# Design, Synthesis, and Biological Evaluation of a Novel Series of 4-Guanidinobenzoate Derivatives as Enteropeptidase Inhibitors with Low Systemic Exposure for the Treatment of Obesity

**DOI:** 10.1021/acs.jmedchem.2c00463

**Published:** 2022-06-10

**Authors:** Zenichi Ikeda, Keiko Kakegawa, Fumiaki Kikuchi, Sachiko Itono, Hideyuki Oki, Hiroaki Yashiro, Hideyuki Hiyoshi, Kazue Tsuchimori, Kenichi Hamagami, Masanori Watanabe, Masako Sasaki, Youko Ishihara, Kimio Tohyama, Tomoyuki Kitazaki, Tsuyoshi Maekawa, Minoru Sasaki

**Affiliations:** † Research, Takeda Pharmaceutical Company Limited, 26-1, Muraokahigashi 2-chome, Fujisawa, Kanagawa 251-8555, Japan; ‡ Research Division, SCOHIA PHARMA, Inc., 26-1, Muraokahigashi 2-chome, Fujisawa, Kanagawa 251-8555, Japan; § Pharmaceutical Sciences, Takeda Pharmaceutical Company Limited, 26-1, Muraokahigashi 2-chome, Fujisawa, Kanagawa 251-8555, Japan

## Abstract

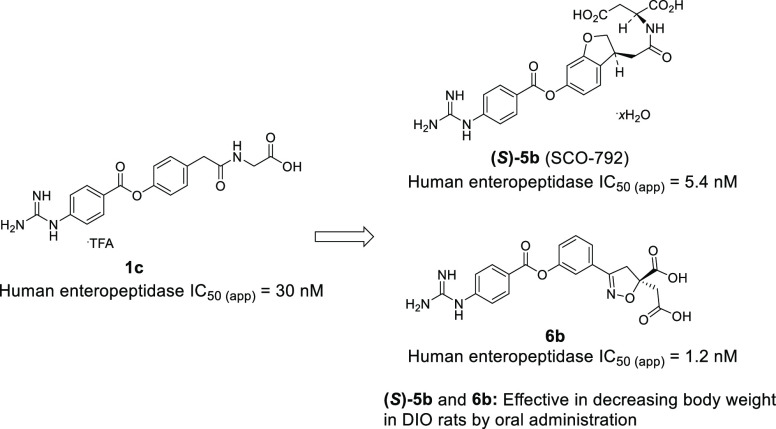

To discover a novel series of potent inhibitors of enteropeptidase, a membrane-bound serine protease localized to the duodenal brush border, 4-guanidinobenzoate derivatives were evaluated with minimal systemic exposure. The **1c** docking model enabled the installation of an additional carboxylic acid moiety to obtain an extra interaction with enteropeptidase, yielding **2a**. The oral administration of **2a** significantly elevated the fecal protein output, a pharmacodynamic marker, in diet-induced obese (DIO) mice, whereas subcutaneous administration did not change this parameter. Thus, systemic exposure of **2a** was not required for its pharmacological effects. Further optimization focusing on the in vitro IC_50_ value and *T*_1/2_, an indicator of dissociation time, followed by enhanced in vivo pharmacological activity based on the ester stability of the compounds, revealed two series of potent enteropeptidase inhibitors, a dihydrobenzofuran analogue (**(*S*)-5b**, SCO-792) and phenylisoxazoline (**6b**), which exhibited potent anti-obesity effects despite their low systemic exposure following their oral administration to DIO rats.

## Introduction

Obesity is a risk factor for lifestyle-related diseases (e.g., type 2 diabetes, hypertension, and dyslipidemia), cardiovascular diseases (e.g., heart failure, myocardial infarction, and stroke), and other disorders.^1,2^ Although diet and exercise are standard treatments, their body weight-lowering effect is limited, and supplementary treatment is required to achieve further body weight loss.^3^ Currently, there are several drugs approved for the treatment of obesity.^4,5^ Among them, central nervous system-acting drugs, such as the combination of phentermine and topiramate and of bupropion and naltrexone, have not fully met the medical needs owing to concerns regarding their efficacy and safety.^6,7^ As a peripheral anti-obesity drug, the lipase inhibitor orlistat has been approved for use.^5^ Although orlistat causes total body weight loss,^8^ mechanism-related gastrointestinal unfavorable effects, such as oily spotting, fecal incontinence, and diarrhea, are observed.^9^ Recently, bariatric surgery, such as Roux-en-Y, sleeve gastrectomy, and gastric banding, are emerging as powerful options to achieve significant body weight loss.^10,11^ However, bariatric surgery is invasive and places a heavy burden on patients. Therefore, the development of safe and noninvasive anti-obesity treatments is highly desired.

**Figure 1:**
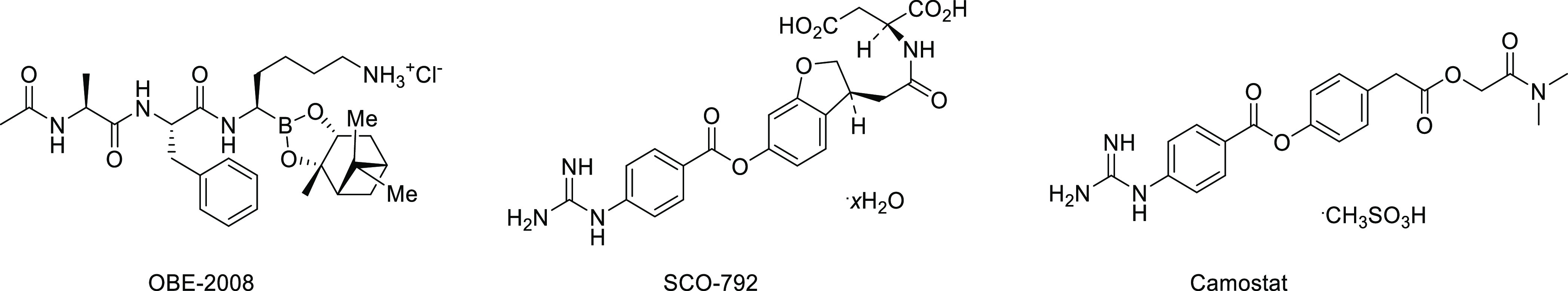
Small-molecule enteropeptidase inhibitors that exert pharmacological effects in DIO mice.

Enteropeptidase (enterokinase, EC3.4.21.9) is a membrane-bound serine protease found in the duodenal lumen, which plays an important role in dietary protein digestion by the conversion of trypsinogen into active trypsin.^12^ After activation, trypsin further activates other zymogens, including chymotrypsinogen, proelastase, and procarboxypeptidases, which leads to the absorption of amino acids.^13^ Notably, congenital deficiency of enteropeptidase in humans results in a lean phenotype.^14,15^ Furthermore, several small-molecule enteropeptidase inhibitors have been reported to exert pharmacological effects in diet-induced obese (DIO) mice (Figure 1).^15−17^

OBE-2008, a borolysine analogue, significantly reduced the rate of body weight gain during the growth phase in DIO mice.^15^ SCO-792, a 4-guanidinobenzoate derivative, is reported to exhibit potent body weight reduction in DIO mice.^16^ Another example of a 4-guanidinobenzoate analogue, camostat, which is an inhibitor of trypsin-like serine proteases, inhibited enteropeptidase activity and induced body weight loss in DIO mice.^17^ These findings suggest that the inhibition of enteropeptidase activity may be an effective treatment for obesity. In this study, we describe medicinal chemistry efforts to identify a novel series of 4-guanidinobenzoate derivatives as enteropeptidase inhibitors, including a dihydrobenzofuran analogue (SCO-792) and a phenylisoxazoline analogue.

Because enteropeptidase is predominantly expressed on the surface of the duodenal lumen, inhibitors of enteropeptidase do not require systemic exposure for pharmacological efficacy. As the avoidance of the systemic circulation of drugs generally lowers the risk of side effects, potent enteropeptidase inhibitors with low systemic exposure are expected to be promising therapeutic agents with minimal risk of adverse effects.

Enteropeptidase is known to recognize the lysine residue of its substrate, namely, trypsinogen, with Asp965 at the S1 site.^18,19^ Thus, we hypothesized that compounds with basic moieties, such as amino, amidyl, and guanidyl groups, could mimic the lysine residue of the substrate, leading to potent inhibition of enteropeptidase. Several strategies have been reported for the discovery of drug candidates with low systemic exposure, including lowering membrane permeability, utilization of active efflux via transporters, and induction of metabolism in the circulation system.^20−22^ In this context, strong basic moieties in enteropeptidase inhibitors can be expected to contribute to low membrane permeability due to their ionized state under physiological conditions, which encouraged us to investigate potent inhibitors of enteropeptidase with low systemic exposure by lowering membrane permeability.

## Results and Discussion

**Figure 2:**
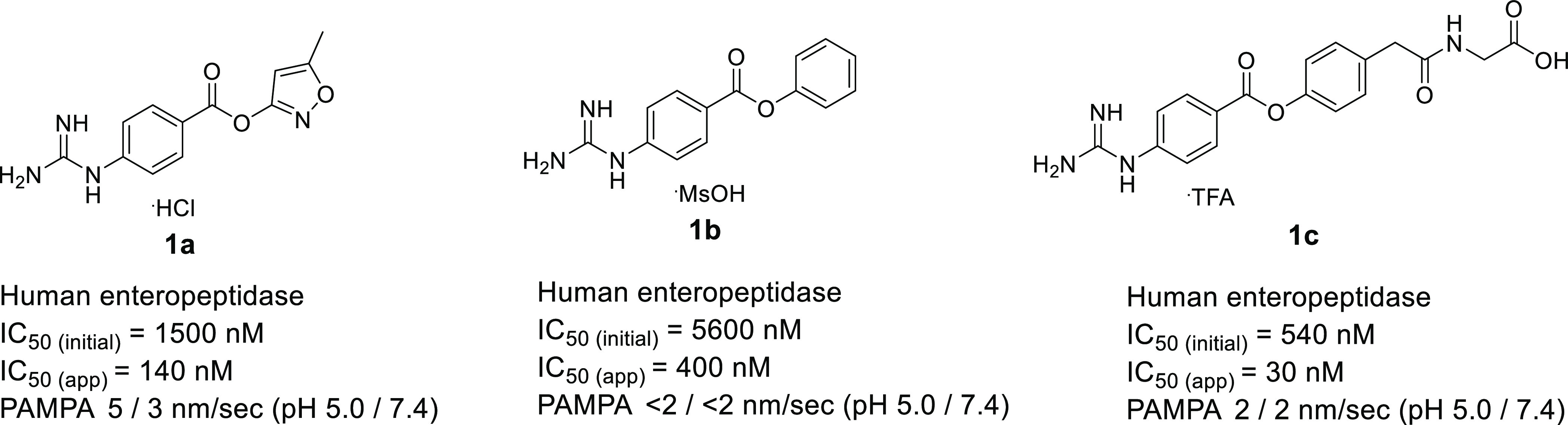
Structures of **1a–1c**. Human enteropeptidase IC_50(initial)_ refers to the inhibitory activity of human enteropeptidase after 6 min of incubation with the enzyme, substrate, and compound. Human enteropeptidase IC_50(app)_ refers to the apparent IC_50_ value after 120 min of incubation with the enzyme, substrate, and compound.

**Figure 3:**
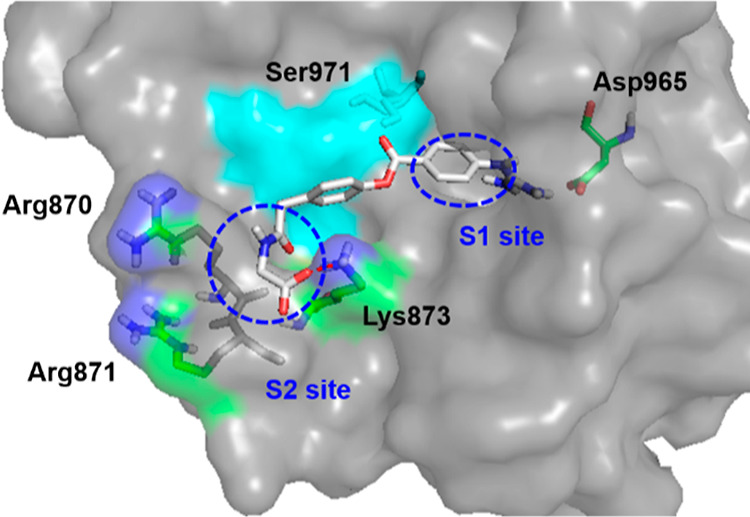
Docking model of **1c** with enteropeptidase. The apo form of human enteropeptidase (PDB ID: 4DGJ) was used as a template. The surface of the catalytic triad (His825, Asp876, and Ser971) is illustrated in cyan.

High-throughput screening of a series of amidine/guanidine compounds led to the identification of phenyl 4-guanidinobenzoate **1a** and **1b** as hit compounds (Figure 2). The guanidinyl group of **1a** and **1b** was expected to mimic the lysine residue of the substrate, thereby leading to enteropeptidase inhibition. Because enteropeptidase cleaves trypsinogen after the sequence Asp–Asp–Asp–Asp–Lys,^18^ incorporation of a carboxylic acid moiety could mimic the aspartic acid residue of the substrate to enhance enteropeptidase inhibitory activity, leading to design **1c**. As shown in Figure 2, **1c** exhibited more potent enteropeptidase inhibitory activity than that of **1b**. To confirm the hypothesis, a docking model of **1c** was constructed according to the reported X-ray crystal structure of the apo form of human enteropeptidase (PDB ID: 4DGJ; Figure 3). As expected, the guanidinyl group was recognized by Asp965 at the S1 site, which was consistent with the results of a previous report.^17^ Furthermore, the carboxylic acid moiety of **1c** interacted with Lys873 at the S2 site, demonstrating the enhanced enteropeptidase inhibitory activity of **1c** compared to **1b**.

**Figure 4:**
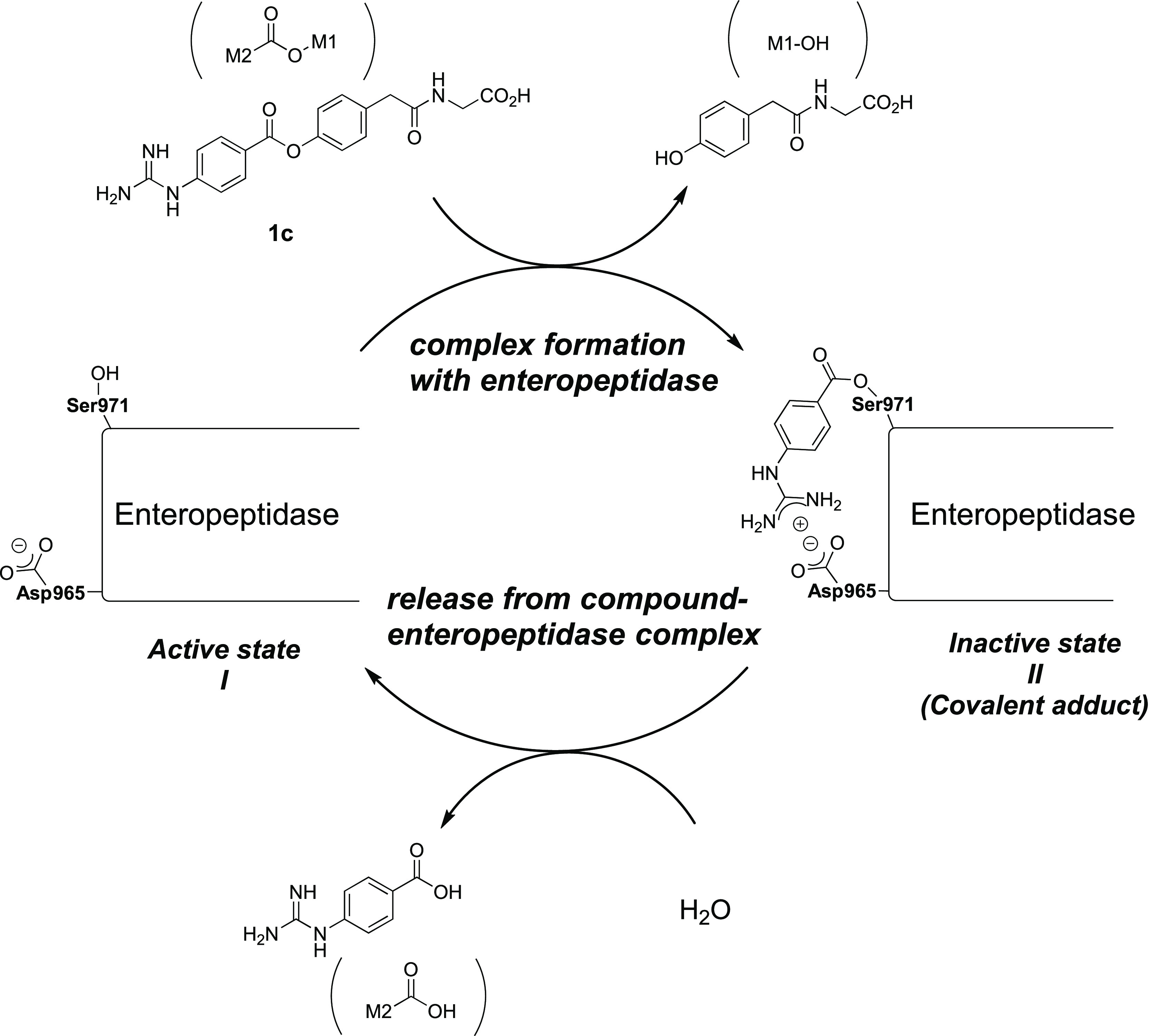
Proposed mechanism for enteropeptidase inhibition by **1c**.

As shown in Figure 2, **1a–1c** were confirmed to inhibit enteropeptidase in a time-dependent manner. Based on the chemical structure, these analogues should covalently inhibit enteropeptidase.^23^ These considerations have been established by recent reports, which revealed that the 4-guanidinobenzoates were reversible covalent inhibitors of enteropeptidase by forming the acyl–enzyme complex via catalytic serine with ionic interaction between the guanidinyl group and the aspartic acid residue in the S1 site.^17,24^ Owing to these findings, we envisaged the mechanism for enteropeptidase inhibition by **1c** shown in Figure 4, which includes the following steps: (a) complex formation with enteropeptidase and (b) release from the compound-enteropeptidase complex. Thus, in the complex formation with the enteropeptidase step, active enteropeptidase (**I**) recognizes inhibitor **1c**, forming a covalent bond that leads to the inactive state (**II**). Thereafter, in the release from compound-enteropeptidase complex step, the covalent adduct undergoes hydrolysis to regenerate active enteropeptidase (**I**). As enteropeptidase is expressed in the duodenal lumen, inhibitors have limited time of action and transit time. Therefore, based on the proposed mechanism, we hypothesized that the acceleration of the complex formation with the enteropeptidase step and slowing down of the release from the compound-enteropeptidase complex step would prolong the lifetime of inactive enteropeptidase (**II**), leading to enhanced pharmacological activities in the duodenal lumen. In general, covalent inhibitors are disfavored as drug classes as they can exhibit off-target activity, which causes undesirable adverse effects. However, reversible covalent inhibitors are a more attractive class of drugs as they can exhibit sufficient efficacy without severe adverse effects by avoiding permanent binding of the inhibitor and target protein.^25^ Furthermore, for enteropeptidase, the duodenal lumen is the predominant site of action. Therefore, even for reversible covalent inhibitors, both potent pharmacological effects and sufficient safety could be achieved by lowering their systemic exposure. In fact, **1a–1c** showed extremely low membrane permeability most likely due to their ionized state under physiological conditions, suggesting that these compounds are promising starting points for potent and safe drug candidates.

**Figure 5:**
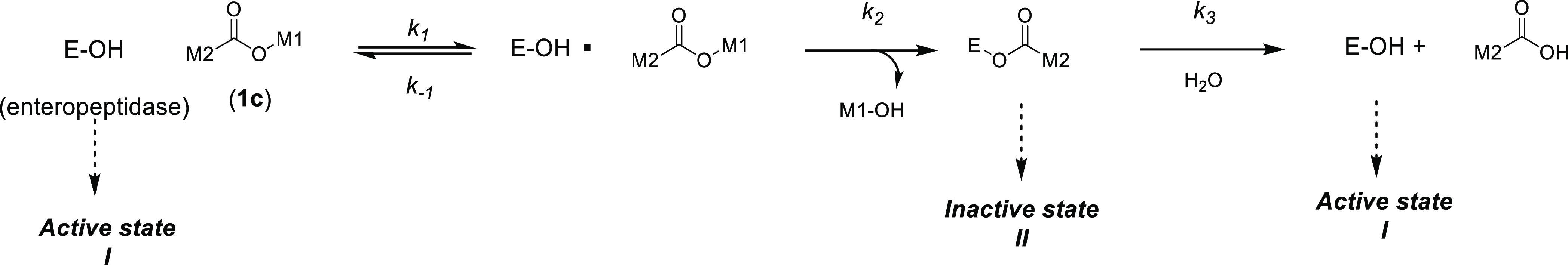
Inhibitory reaction. In this equation, *k*_1_ and *k*_–1_ are rate constants of association and dissociation of enzyme and inhibitor and *k*_2_ and *k*_3_ are the reaction rate constants of acylation and deacylation, respectively.

The inhibitory reaction can be described as shown in Figure 5,^26^ where *k*_1_, *k*_–1_, and *k*_2_ contribute to the complex formation with the enteropeptidase step and *k*_3_ contributes to the release from the compound-enteropeptidase complex step. Although not all reaction constants for **1c** could be identified, the in vitro IC_50_ value could be used as an indicator of the combination of *k*_1_, *k*_–1_, and *k*_2_. In this case, the IC_50_ value of a compound is derived from its affinity for enteropeptidase (*k*_1_ and *k*_–1_) and its reactivity against enteropeptidase (*k*_2_). In the course of compound optimization, enhancing the affinity to enteropeptidase instead of increasing the reactivity is a preferable strategy for obtaining more potent and safe compounds by lowering the risk of undesirable off-target activities. As the affinity of a compound for enteropeptidase is expected to largely contribute to the IC_50_ value based on initial velocities (IC_50(initial)_) relative to the IC_50_ value based on the steady state (IC_50(app)_), we performed chemical modifications based on IC_50(initial)_. In addition, we evaluated the regeneration rate of active enteropeptidase (**I**) from the inactive state (**II**) using an in vitro dissociation assay, which measured the recovery rate (*T*_1/2_) of active enteropeptidase by rapid dilution of the assay media after 2 h of preincubation of the test compound and enteropeptidase at high concentrations. Because *T*_1/2_ can be an indicator of *k*_3_, we focused on compounds with low IC_50(initial)_ values and long *T*_1/2_ values.

Based on the docking model of **1c** (Figure 3), we attempted to increase the enteropeptidase inhibitory activity by inducing an extra interaction between **1c** and enteropeptidase. We focused on other basic residues in the S2 site, such as Arg870 and/or Arg871, as further replacement of the glycine part in **1c** with acidic amino acids could lead to an interaction with arginine residues, resulting in an increase in potency. In addition, the introduction of a charged carboxyl group can contribute to lower membrane permeability.

**Figure 6:**
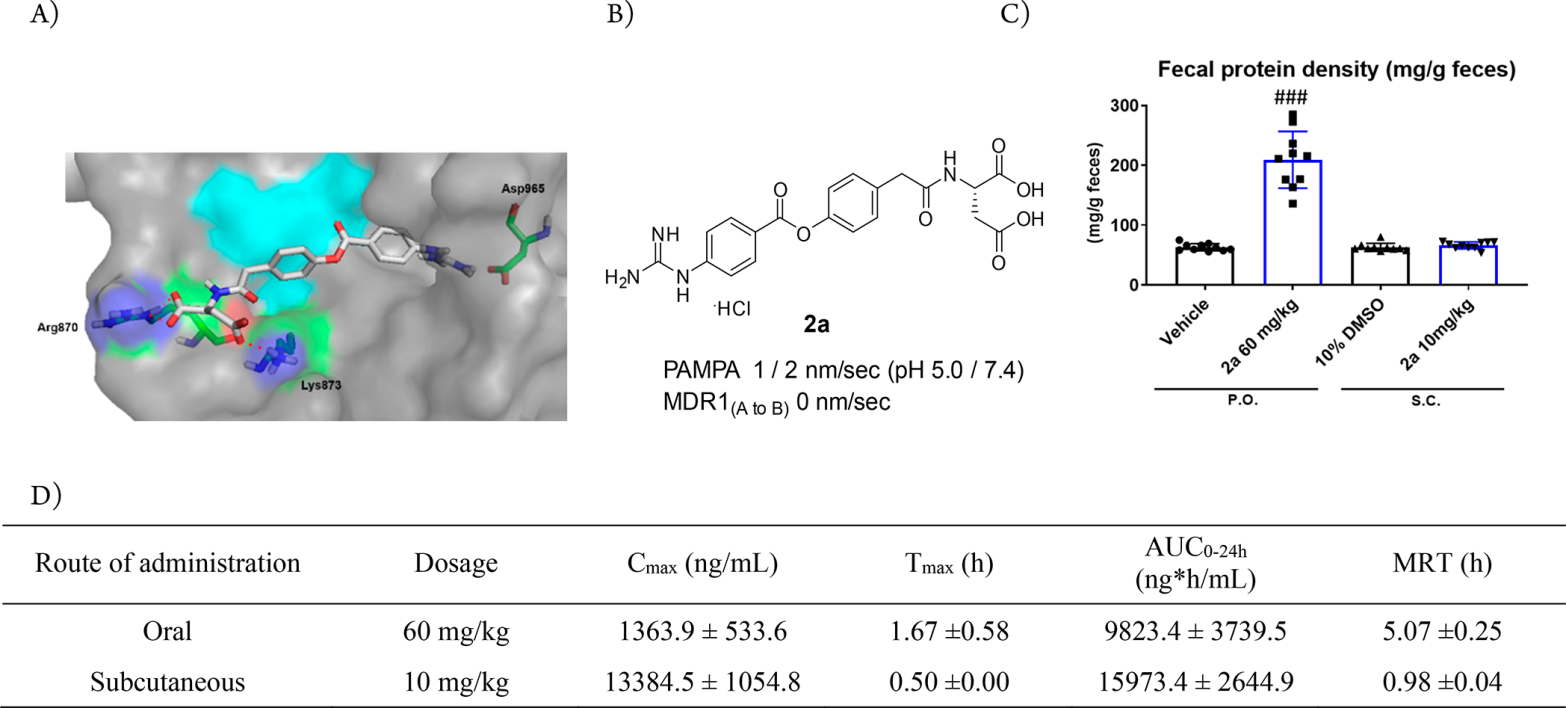
(A) Docking model of **2a** with enteropeptidase. The apo form of human enteropeptidase (PDB ID: 4DGJ) was used as a template. The surface of the catalytic triad is highlighted in cyan. (B) Properties of **2a**. (C) Increase in fecal protein output. (D) Plasma exposure of **2a** by oral (60 mg/kg) and subcutaneous (10 mg/kg) administration in mice. ###; *P* ≤ 0.01, vs vehicle using the Aspin–Welch test vs the vehicle.

The inhibitory activity of the synthesized compounds against human enteropeptidase was evaluated. As shown in Table 1, replacement of the glycine moiety of **1c** with aspartic acid resulted in a five-fold enhancement in IC_50(initial)_ (**2a**), whereas the asparagine derivative **2b** slightly improved its initial inhibitory activity. Installation of glutamic acid also led to an increase in enteropeptidase inhibitory activity (**2c**). These results suggest that the additional carboxyl groups of **2a** and **2c** afforded an extra interaction with enteropeptidase, thereby enhancing the inhibitory activity. Figure 6A illustrates a docking model of **2a** with enteropeptidase and reveals the interaction of the two carboxyl groups with Arg870 and Lys873. As shown in Figure 6B, **2a** exhibited low membrane permeability, as expected. To confirm whether **2a** can inhibit enteropeptidase activity in vivo, a pharmacodynamic study was performed. Considering the role of enteropeptidase in protein digestion, we hypothesized that enteropeptidase inhibition in vivo would result in the elevation of protein levels in feces. As expected, **2a** exhibited a significant increase in fecal protein content compared to the vehicle after oral administration in mice (Figure 6C). However, subcutaneous administration of **2a** at a dose that covers a higher AUC than that of oral administration did not increase fecal protein levels (Figure 6C,D). These results clearly demonstrate that systemic exposure to **2a** was not required for pharmacological efficacy, supporting our strategy to target duodenal enteropeptidase with potent inhibitors that exhibit low systemic exposure. Based on these promising data, **2a** was employed as a lead compound, and lead optimization was initiated to enhance its pharmacological activity.

**Table 1:**
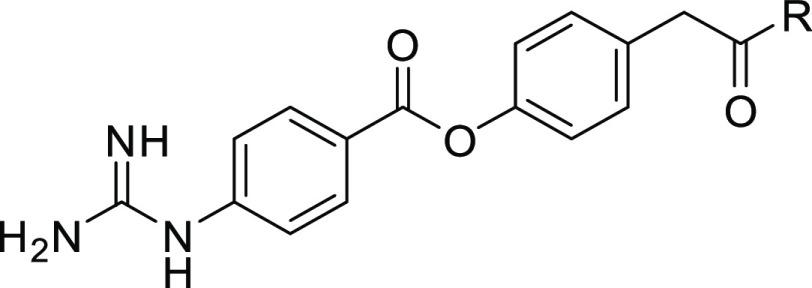

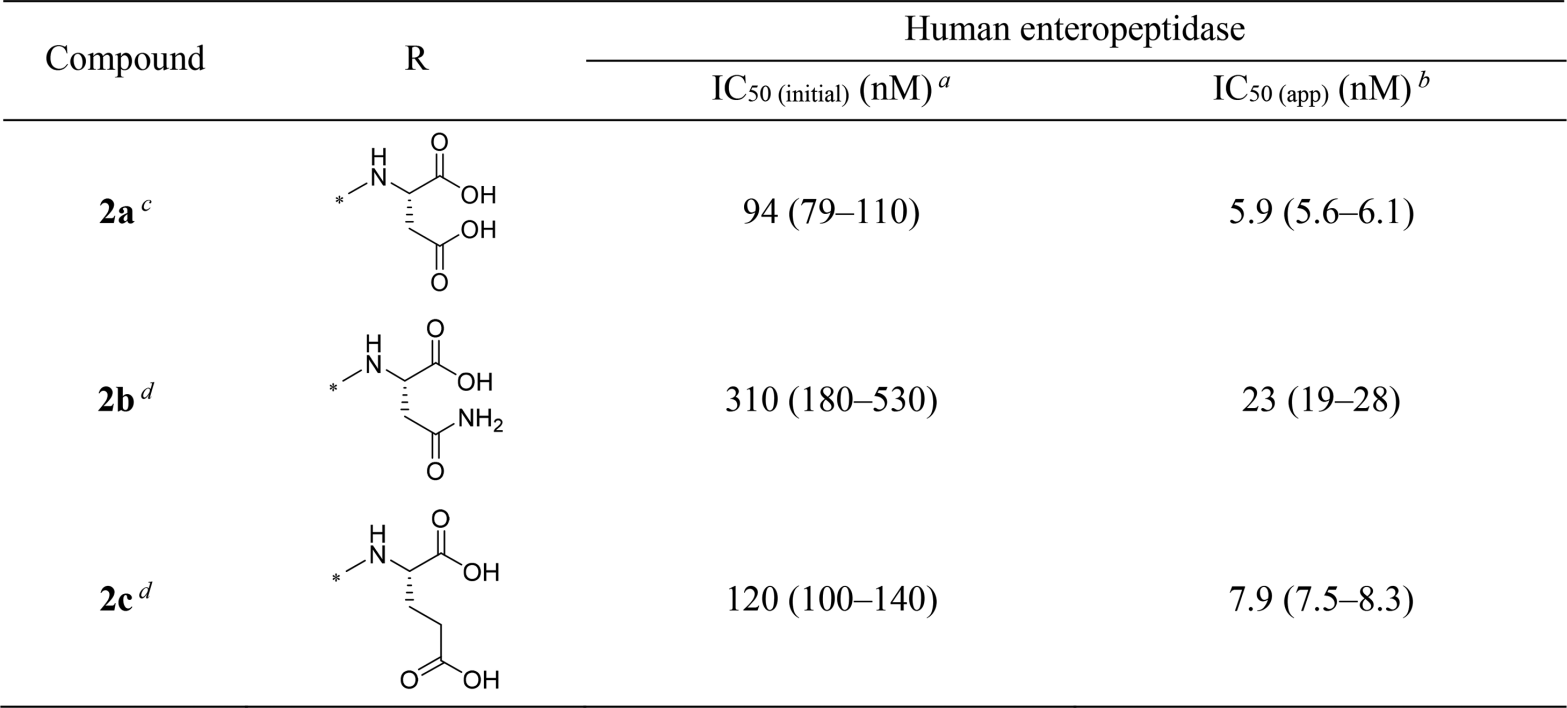
In Vitro Activities of **2a–c**

a Inhibitory activities of compounds against human enteropeptidase. The assay was carried out by incubating human enteropeptidase, substrate, and compound at room temperature for 6 min (for IC_50(initial)_). The IC_50_ values are presented with 95% confidence intervals in parentheses.

b Inhibitory activities of compounds against human enteropeptidase. The assay was carried out by incubating human enteropeptidase, substrate, and compound at room temperature for 120 min (for IC_50(app)_). The IC_50_ values are presented with 95% confidence intervals in parentheses.

c HCl salt.

d TFA salt.

Assuming that *T*_1/2_ and the IC_50(initial)_ values were markedly influenced by the steric and/or electronic effects of the substituent on the benzene ring on the left-hand side (LHS), we first determined the substituent effects at 2- and 3-position on the LHS benzene ring; the results are presented in Table 2. Installation of a methyl group at the 3-position resulted in more than 30-fold decrease in IC_50(initial)_ (**3a**), which could be explained by the extremely narrow space around the 3-position, as observed in Figure 6A. By contrast, the 2-methyl analogue **3b** showed more potent activity than the 3-substituted derivative **3a**. The docking model of **2a** (Figure 6A) suggests that a small substituent could be accommodated around the 2-position. In this case, the electron-donating or steric effect of the 2-methyl group would most likely hinder the reactivity of the ester bond with enteropeptidase, causing a decrease in the inhibitory activity compared with **2a**. Therefore, we evaluated the effect of the introduction of an electron-withdrawing group at the 2-position. Incorporating a chlorine atom (**3c**) enhanced the enteropeptidase inhibitory activity to a level comparable to that of **2a**; this could be attributed to the electron-withdrawing effect of the chlorine atom, which increased the reactivity of the ester bond. Thus, IC_50(initial)_ is likely to include reactivity against enteropeptidase as well as affinity to enteropeptidase. Consequently, we installed a fluorine atom at the 2-position as a smaller and stronger electron-withdrawing substituent. As expected, **3d** showed significantly enhanced inhibitory activity compared to that of **2a**. Moreover, the introduction of an electron-withdrawing chlorine (**3c**) or fluorine atom (**3d**) resulted in a decrease in *T*_1/2_, whereas the *T*_1/2_ value of the methyl-substituted analogue **3b** was off-scale high similar to the non-substituted lead **2a**. These results are consistent with the enteropeptidase inhibition mechanism shown in Figure 4. Thus, the electron-withdrawing substituents at the 2-position destabilize the ester bond of the covalent adduct (**II**), causing an increase in the recovery rate of the active enteropeptidase. The introduction of electron-withdrawing substituents had a negative effect on *T*_1/2_ and a positive effect on the IC_50(initial)_ value. Collectively, these results suggest that non-substituted benzene is the best choice for the LHS benzene ring in terms of both IC_50(initial)_ and *T*_1/2_.

**Table 2:**
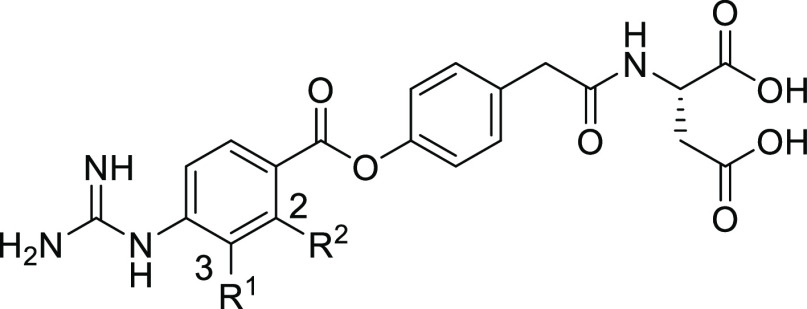
In Vitro Activities and *T*_1/2_ of **2a** and **3a–d**

			human enteropeptidase		
compound	R^1^	R^2^	IC_50(initial)_ (nM)^a^	IC_50(app)_ (nM)^b^	*T*_1/2_ (min)^c^
**2a**^d^	H	H	94 (79–110)	5.9 (5.6–6.1)	>120
**3a**	Me	H	>3300	680 (600–770)	NT^e^
**3b**	H	Me	940 (750–1200)	54 (50–58)	>120
**3c**	H	Cl	57 (45–72)	5.6 (5.4–5.9)	14
**3d**	H	F	7.4 (6.1–8.8)	0.81 (0.78–0.85)	6.1

a Inhibitory activities of compounds against human enteropeptidase. The assay was carried out by incubating human enteropeptidase, substrate, and compound at room temperature for 6 min (for IC_50(initial)_). The IC_50_ values are presented with 95% confidence intervals in parentheses.

b Inhibitory activities of compounds against human enteropeptidase. The assay was carried out by incubating human enteropeptidase, substrate, and compound at room temperature for 120 min (for IC_50(app)_). The IC_50_ values are presented with 95% confidence intervals in parentheses.

c Half-time of dissociation from human enteropeptidase.

d HCl salt.

e Not tested.

We proceeded to optimize the position of the aspartic acid amide side chain on the right-hand side (RHS) benzene ring of **2a** and its linker length to achieve a lower IC_50(initial)_ value (Table 3). In terms of the substituent position, the 3-substituted analogue **4a** showed a lower IC_50(initial)_ value than the 4-substituted derivative (**2a**). Furthermore, removal of the methylene linker resulted in a markedly lower IC_50(initial)_ value (**4b**). Notably, the electron-withdrawing effect of the amide moiety directly attached to the RHS benzene ring increased the reactivity of the ester bond of **4b** with enteropeptidase. The same tendency was observed for 4-substituted analogue **4c**, showing a lower IC_50(initial)_ than **2a**. **4b**, bearing the amide moiety directly attached to the RHS benzene ring, exhibited the lowest IC_50(initial)_ value.

**Table 3:**
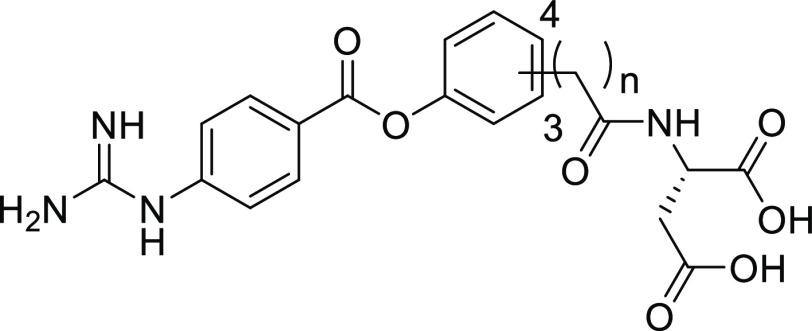
In Vitro and In Vivo Activities of **4a–c**

			human enteropeptidase			
compound	*n*	substituent position	IC_50(initial)_ (nM)^a^	IC_50(app)_ (nM)^b^	fecal protein output (fold of **2a**)^c^	stability at pH 1.2/6.8 (% decomposed)^d^
**2a**^e^	1	4	94 (79–110)	5.9 (5.6–6.1)	(1.00)	2.5/5.0
**4a**^f^	1	3	32 (26–39)	1.6 (1.5–1.8)	0.97	2.8/5.8
**4b**^f^	0	3	13 (9.6–18)	0.82 (0.67–1.0)	0.82	3.9/8.6
**4c**^f^	0	4	65 (49–86)	3.2 (3.1–3.4)	0.88	NT^g^

a Inhibitory activities of compounds against human enteropeptidase. The assay was carried out by incubating human enteropeptidase, substrate, and compound at room temperature for 6 min (for IC_50(initial)_). The IC_50_ values are presented with 95% confidence intervals in parentheses.

b Inhibitory activities of compounds against human enteropeptidase. The assay was carried out by incubating human enteropeptidase, substrate, and compound at room temperature for 120 min (for IC_50(app)_). The IC_50_ values are presented with 95% confidence intervals in parentheses.

c Compound was orally administered to mice (10 mg/kg).

d % decomposition at 24 h.

e HCl salt.

f TFA salt.

g Not tested.

Using compounds that demonstrated potent effects in vitro, we conducted an in vivo evaluation by measuring proteins in feces as a pharmacodynamic marker after the oral administration of the synthesized compounds in mice (10 mg/kg). As shown in Table 3, the increase in the fecal protein output by **4b** and **4c** was weaker than that of the corresponding methylene-inserted analogues **4a** and **2a**, respectively. These results were contrary to our expectations as compounds with the lowest IC_50_ values were expected to display the most potent increase in fecal protein output in vivo. We hypothesized that this discrepancy between the in vitro and in vivo activities could be explained in terms of the duodenal concentration of the compounds. These compounds showed excellent aqueous solubility under acidic and neutral conditions.^27^ By contrast, a series of synthesized compounds possess the ester and amide moieties, which may be cleaved in the physiological condition through hydrolysis and/or some hydrolytic enzymes after oral administration. In this regard, the ester bonds of **4b** and **4c** were expected to be more fragile than those of **4a** and **2a** because of the electron-withdrawing effect of the directly attached amide moiety. An in vitro aqueous stability test revealed that **4b** was less stable than **4a** and **2a** under acidic and neutral conditions. Although the actual concentration of each compound in the duodenum was not quantified, we hypothesized that the aqueous stability of a compound could be utilized as an indicator of its stability in vivo, thereby affecting its duodenum concentration and pharmacological activity. Thus, we selected **2a** as a suitable lead compound based on its high aqueous stability and the fact that it displayed the highest increase in fecal protein output in vivo.

**Figure 7:**
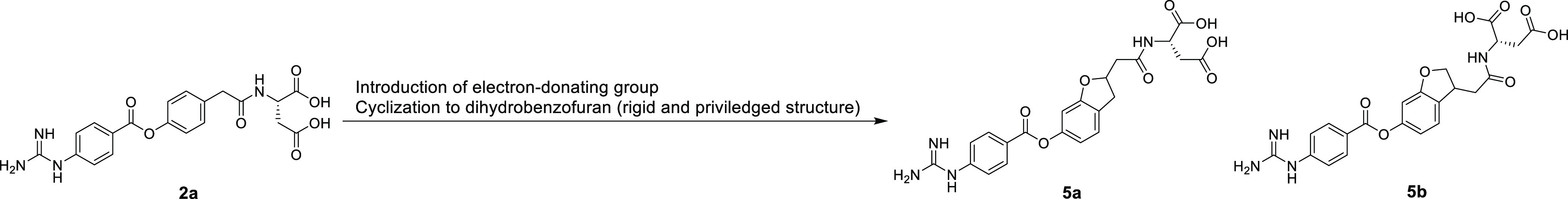
Design of dihydrobenzofuran analogues using intramolecular cyclization.

To further enhance the in vivo activity of **2a**, we optimized its RHS benzene ring. Thus, we considered that the introduction of an electron-donating moiety to the RHS benzene ring would contribute to the stabilization of the ester bond and the subsequent enhancement of protein levels in feces. As an intramolecular cyclization strategy can effectively lock the active conformation of the molecule and the dihydrobenzofuran ring is found in many bioactive compounds,^28,29^ we designed the dihydrobenzofuran analogues **5a** and **5b** (Figure 7). As shown in Table 4, the (*S*)-isomer of 3-substituted dihydrobenzofuran (**(*S*)-5b**) showed the most potent enteropeptidase inhibitory activity among the dihydrobenzofuran analogues, with an IC_50(initial)_ value of 68 nM. Furthermore, **(*S*)-5b** showed better aqueous stability under both acidic and neutral conditions than **2a** as expected. As a result, **(*S*)-5b** exhibited a markedly more potent increase in fecal protein output (1.28-fold of **2a**).

**Table 4:** In Vitro and In Vivo Activities of **5a**, **(*R*)-5b**, and **(*S*)-5b**

	human enteropeptidase			
compound	IC_50(initial)_ (nM)^a^	IC_50(app)_ (nM)^b^	fecal protein output (fold of **2a**)^c^	stability at pH 1.2/6.8 (% decomposed)^d^
**5a**	180 (140–230)	14 (13–16)	NT^e^	NT^e^
**(*R*)-5b**^f^	84 (67–100)	7.7 (7.1–8.3)	1.26	NT^e^
**(*S*)-5b**^g^	68 (49–93)	5.4 (4.7–6.1)	1.28	0.2/4.4
**2a**^h^	94 (79–110)	5.9 (5.6–6.1)	(1.00)	2.5/5.0

a Inhibitory activities of compounds against human enteropeptidase. The assay was carried out by incubating human enteropeptidase, substrate, and compound at room temperature for 6 min (for IC_50(initial)_). The IC_50_ values are presented with 95% confidence intervals in parentheses.

b Inhibitory activities of compounds against human enteropeptidase. The assay was carried out by incubating human enteropeptidase, substrate, and compound at room temperature for 120 min (for IC_50(app)_). The IC_50_ values are presented with 95% confidence intervals in parentheses.

c Compound was orally administered to mice (10 mg/kg).

d % decomposition at 24 h.

e Not tested.

f (*R*)-configuration at the 3-position of dihydrobenzofuran.

g (*S*)-configuration at the 3-position of dihydrobenzofuran.

h HCl salt.

**Figure 8:**
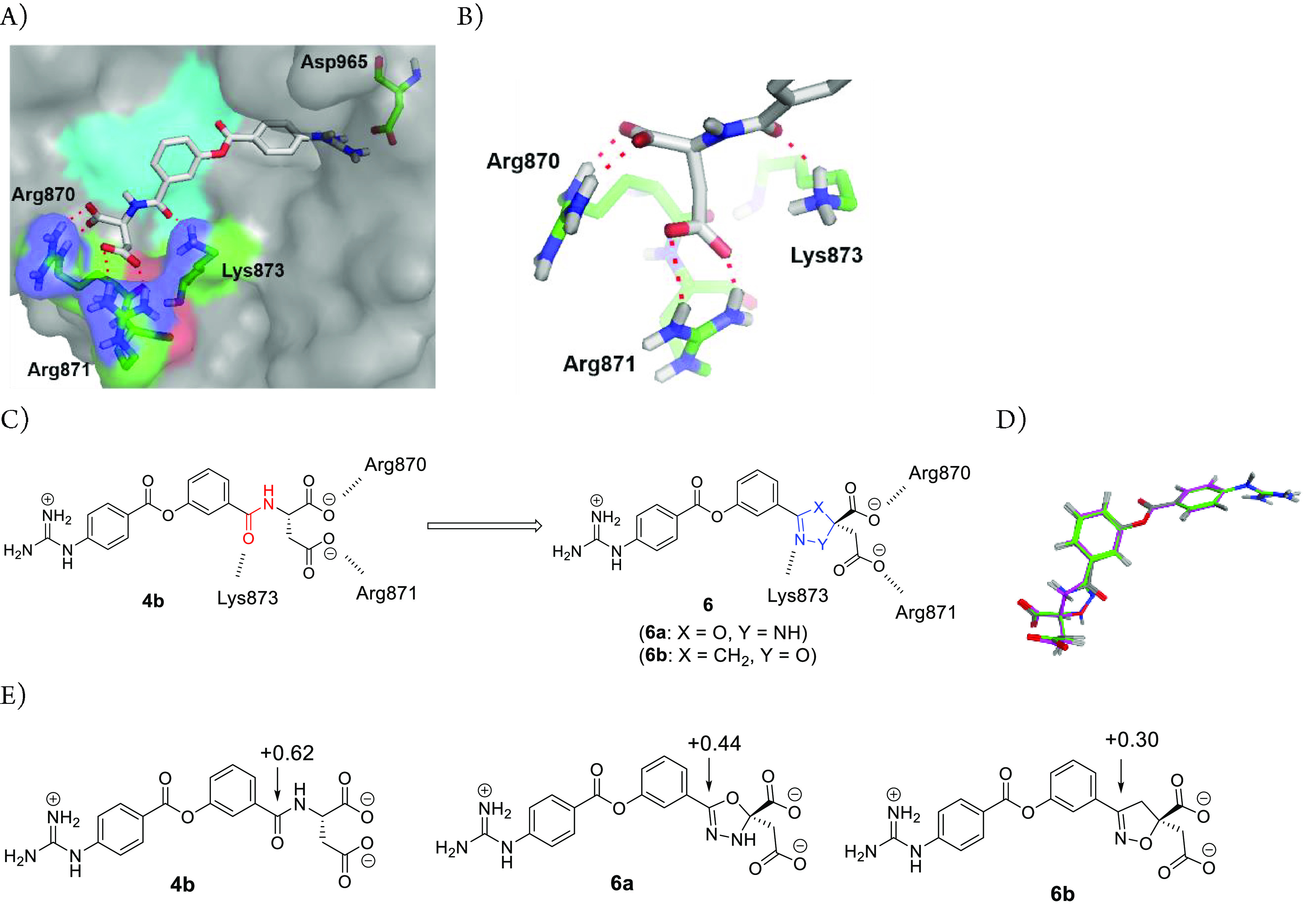
Docking model of **4b** with enteropeptidase. The apo form of human enteropeptidase (PDB ID: 4DGJ) was used as a template. (A) Overall structure. The surface of the catalytic triad is highlighted in cyan. (B) Top view of the S2 site. (C) Replacement of the amide group of **4b** with bioisosteres. (D) Superposition of **6a** (green) and **6b** (magenta) to enteropeptidase-docked **4b** (gray) by MOE.^32^ (E) Calculation of the charge distribution.

We opted to focus on increasing the protein content in feces owing to **4b**, which showed the lowest IC_50(initial)_. We hypothesized that if the aqueous stability of **4b** could be improved without loss of enteropeptidase inhibitory activity, the protein content in feces could be increased in vivo. As the ester moiety of **4b** was relatively unstable due to the directly attached electron-withdrawing amide moiety, we envisioned that a reduction in the electron-withdrawing effect would improve ester stability. Regarding the enteropeptidase inhibitory activity, we considered that the lowest IC_50(initial)_ value of **4b** was due to its enhanced affinity to enteropeptidase as well as increased reactivity. A docking study of **4b** suggested that the dicarboxylic acid moieties in the S2 site form ionic interactions with Arg870 and Arg871. Furthermore, the formation of an additional hydrogen bond between the carbonyl oxygen of the amide moiety and Lys873 could further lower the IC_50(initial)_ value (Figure 8A,B). As reducing the electron-withdrawing effect of the amide while maintaining the key interaction with enteropeptidase might be a suitable approach, we explored alternatives to amides showing weaker electron-withdrawing effects. Because the amide bond itself may be unstable under physiological conditions, replacement of the amide moiety could be a reasonable strategy. Because the aspartic acid amide moiety of **4b** is involved in important interactions at the S2 site, as described in Figure 8A,B, candidates as alternative groups should maintain the ionic interactions of the dicarboxylic acid moiety, maintain the hydrogen bonding of the carbonyl oxygen atom, and reduce the electron-withdrawing effect.

As amide bioisosteres meet the abovementioned requirements, we focused on partially unsaturated five-membered rings containing a nitrogen atom (Figure 8C).^30,31^ We designed 5-phenyloxadiazoline **6a** and 3-phenylisoxazoline **6b** bearing a dicarboxylic acid moiety as candidates for stable isolation. Figure 8D shows the superposition of **6a** and **6b** with enteropeptidase-docked **4b**, revealing a good overlap in both carboxylic acid moieties. Furthermore, the nitrogen atoms of **6a** and **6b** overlapped with the carbonyl oxygen atoms of **4b**. To evaluate the electron-withdrawing effect, we calculated the charge distributions of **6a** and **6b**, focusing on the partial positive charge of the carbon atom attached to the RHS benzene ring, which was determined to be +0.62 for **4b** (Figure 8E). In silico analysis revealed that the carbon atom of **6b** exhibited the least positive charge (+0.30), suggesting a weaker electron-withdrawing effect for the isoxazoline ring. Therefore, **6b** was expected to maintain a high affinity for enteropeptidase while improving its in vivo stability by stabilizing the ester bond and circumventing the possible metabolic cleavage risk of the amide bond, which would lead to enhanced pharmacological effects.

As shown in Table 5, the amide moiety of **4b** was successfully replaced by an isoxazoline ring, resulting in potent enteropeptidase inhibitory activity. An aqueous stability study revealed that (*R*)-isomer **6b** was more stable than that found under acidic and neutral conditions as compared to **4b**. As a result, **6b** boosted the increase in fecal protein output (1.29-fold of **2a**), which could be due to the presence of the isoxazoline ring improving ester bond stability as well as hindering the possible in vivo cleavage of the amide linkage.

**Table 5:**
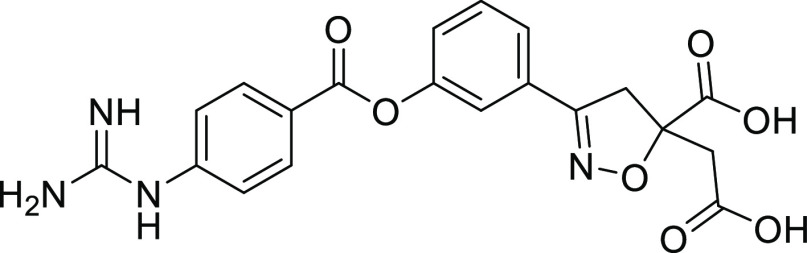
In Vitro and In Vivo Activities of **6b** and **6c**

	human enteropeptidase			
compound	IC_50(initial)_ (nM)^a^	IC_50(app)_ (nM)^b^	fecal protein output (fold of **2a**)^c^	stability at pH 1.2/6.8 (% decomposed)^d^
**6b**^e^	20 (12–33)	1.2 (0.96–1.5)	1.29	1.1/7.3
**6c**^f^	26 (16–43)	1.8 (1.8–1.9)	1.23	NT^g^/NT^g^
**4b**^h^	13 (9.6–18)	0.82 (0.67–1.0)	0.82	3.9/8.6

a Inhibitory activities of compounds against human enteropeptidase. The assay was carried out by incubating human enteropeptidase, substrate, and compound at room temperature for 6 min (for IC_50(initial)_). The IC_50_ values are presented with 95% confidence intervals in parentheses.

b Inhibitory activities of compounds against human enteropeptidase. The assay was carried out by incubating human enteropeptidase, substrate, and compound at room temperature for 120 min (for IC_50(app)_). The IC_50_ values are presented with 95% confidence intervals in parentheses.

c Compound was orally administered to mice (10 mg/kg).

d % decomposition at 24 h.

e (*R*)-configuration at the 5-position of isoxazoline.

f (*S*)-configuration at the 5-position of isoxazoline.

g Not tested.

h TFA salt.

**Figure 9:**
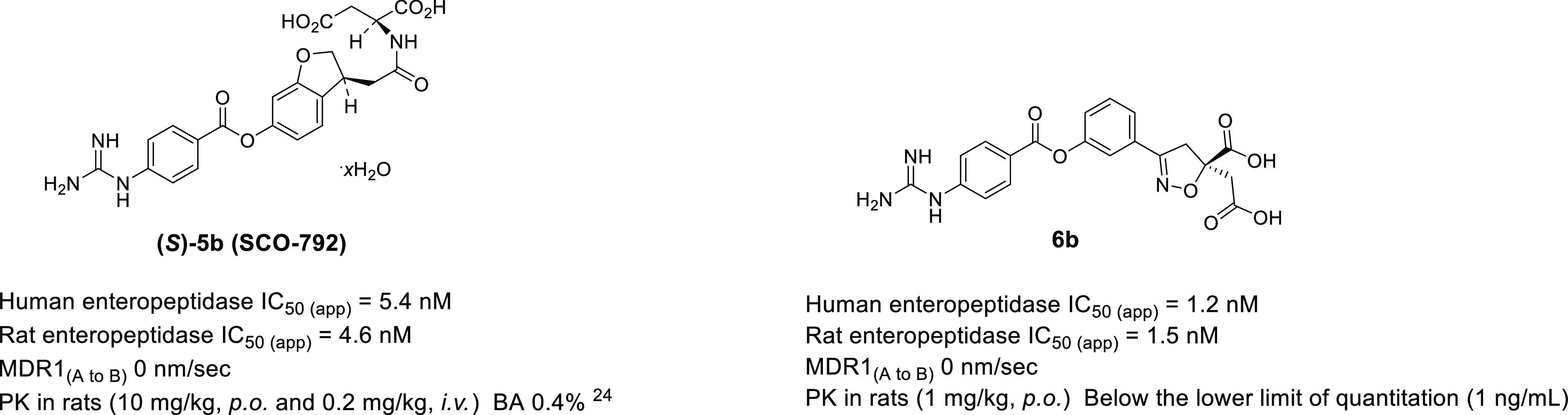
Properties of **(*S*)-5b** and **6b**. Rat enteropeptidase IC_50(app)_ refers to the inhibitory activity of rat enteropeptidase after 120 min of incubation of the enzyme, substrate, and compound.

**Figure 10:**
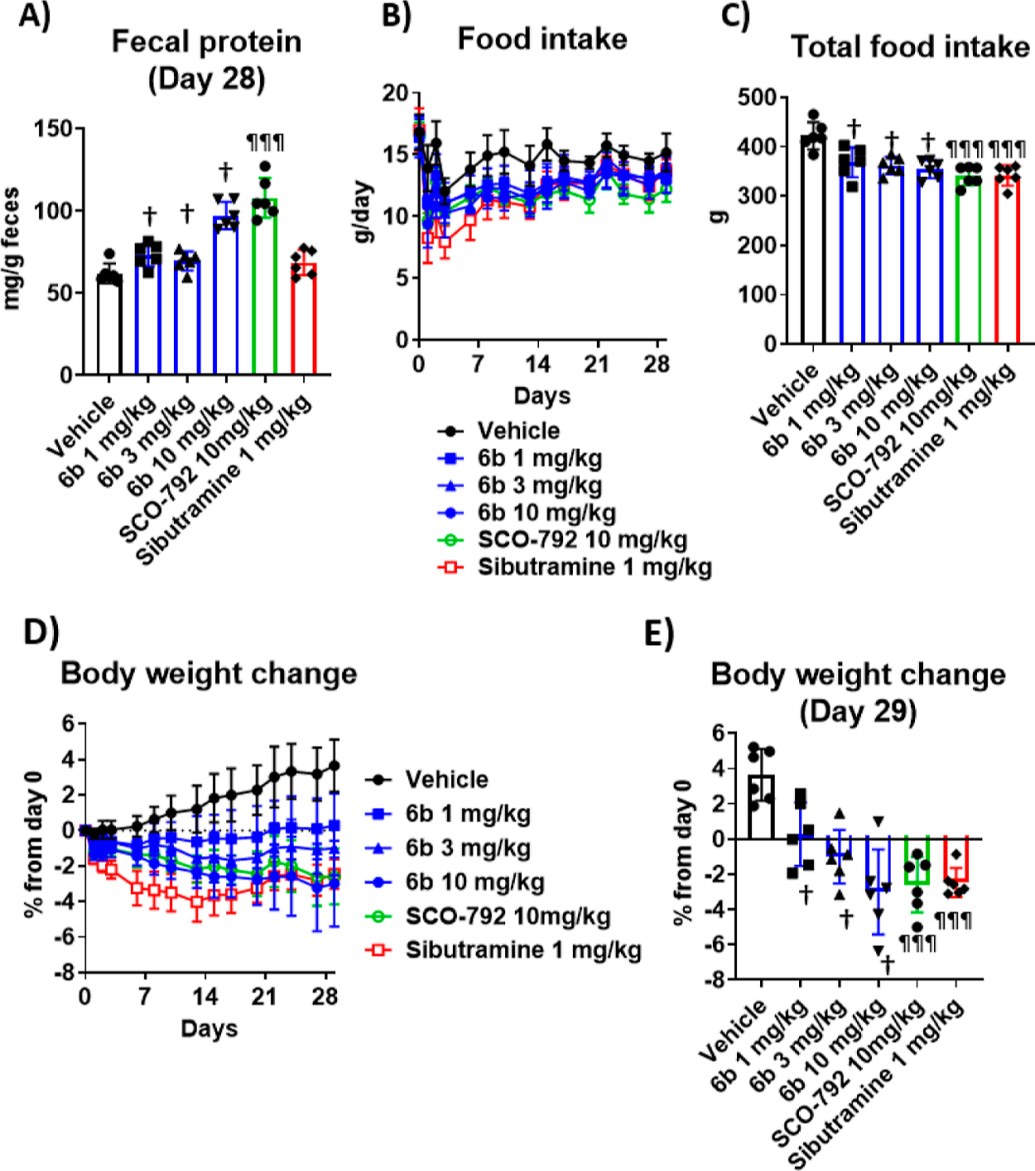
Pharmacological effects by repeated dosing of **(*S*)-5b** (SCO-792) and **6b** in DIO rats. (A) Average fecal protein output on day 28. (B) Food intake during the study. (C) Total food intake. (D) Body weight change from day 0 during the study. (E) Body weight change during the study. Baseline body weight was 498 g. Sibutramine was selected as a control drug as this agent was demonstrated to be effective in both humans^33^ and mice (data not shown) at similar dosage. †; *P* ≤ 0.025 vs vehicle using the one-tailed Williams’ test. ^¶¶¶^; *P* ≤ 0.001, vs vehicle using the Dunnett’s *t*-test. Data are presented as the mean ± SD (*n* = 6 for each group).

We identified two novel series of enteropeptidase inhibitors, dihydrobenzofuran and phenylisoxaziline analogues, that showed potent fecal protein output. As shown in Figure 9, **(*S*)-5b** and **6b** showed potent inhibitory activity against both human and rat enteropeptidases. Furthermore, they displayed extremely low permeability. Pharmacokinetic studies with rats revealed very low or no systemic exposure to either compounds after oral administration. The apparent differences in oral bioavailability were attributed to the dose and detection limits of bioanalysis. We believe that minimal systemic exposure can minimize the risk of side effects. An anti-obesity test was conducted via the oral administration of **(*S*)-5b** and **6b** to DIO rats for four weeks. **(*S*)-5b** showed significant elevation of fecal protein output, reduction of food intake, and body weight loss at 10 mg/kg in DIO rats (Figure 10), consistent with its pharmacological effects in DIO mice.^16^ Likewise, **6b** exhibited potent body weight loss in a dose-dependent manner, along with a significant increase in the fecal protein output and significant reduction in food intake. These results demonstrate the potential of a novel series of enteropeptidase inhibitors for obesity treatment.

## Chemistry

**Scheme 1:**
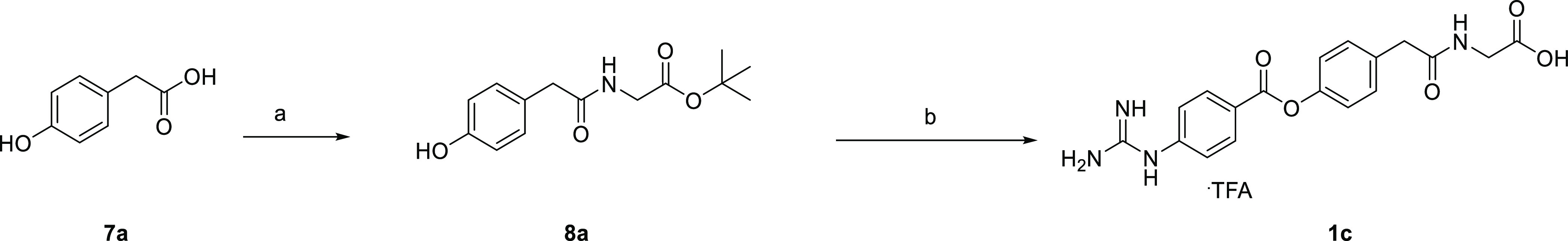
Synthesis of **1c** Reagents and conditions: (a) glycine *tert*-butyl ester hydrochloride, WSC, HOBt, Et_3_N, DMF, rt, overnight, 96%; (b) (1) 4-carbamimidamidobenzoyl chloride hydrochloride, pyridine, acetonitrile, rt, overnight, (2) 4 M HCl/EtOAc, rt, 4 h, then, TFA, rt, 1 h, 30%.

Scheme 1 describes the synthesis of **1c**. Amidation reaction of carboxylic acid **7a** with glycine *tert*-butyl ester afforded amide **8a**. Subsequent treatment with 4-carbamimidamidobenzoyl chloride followed by deprotection gave the target compound.

**Scheme 2:**
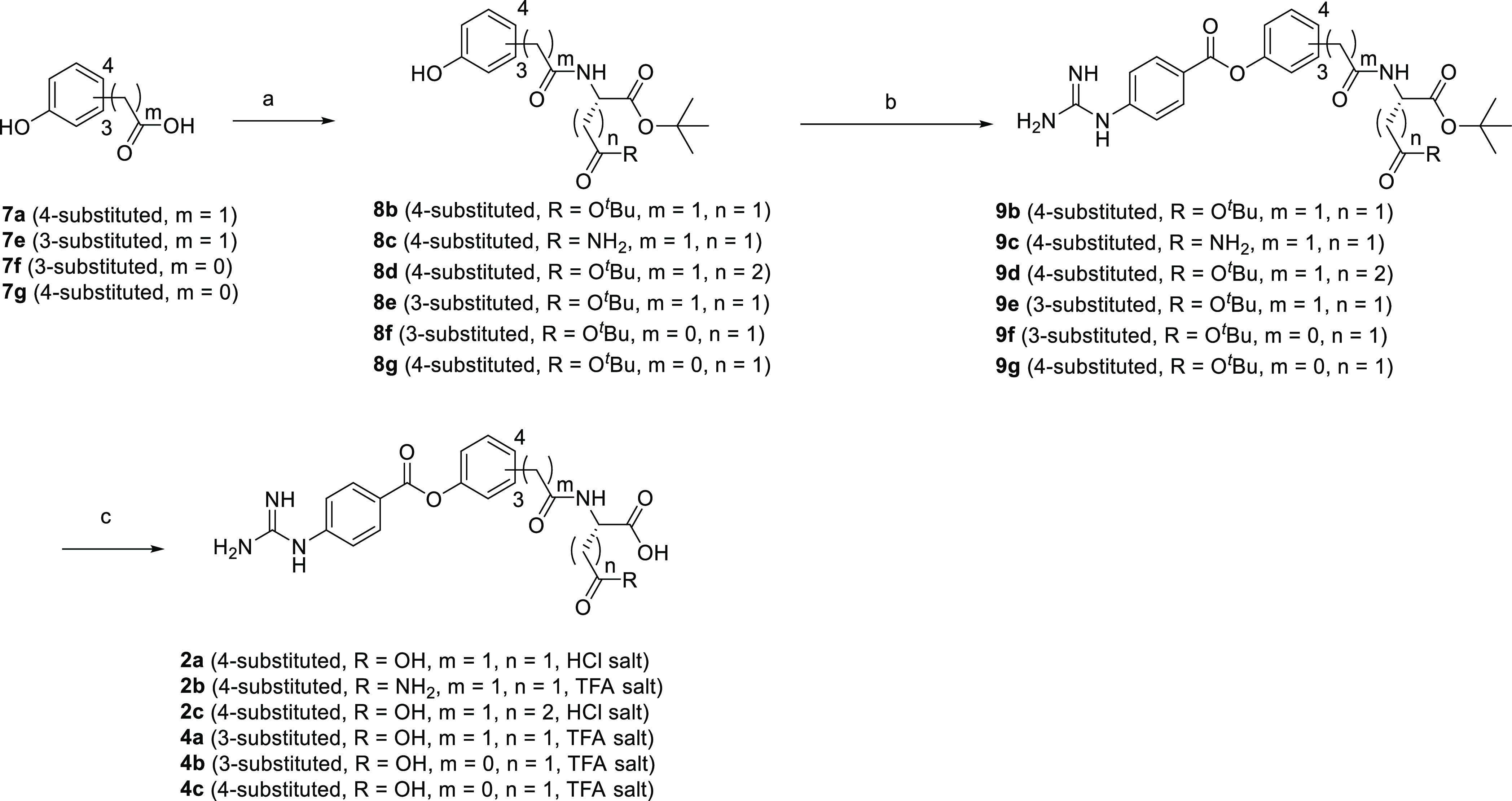
Synthesis of **2a–c** and **4a–c** Reagents and conditions: (a) Boc-protected amino acid, WSC, HOBt, Et_3_N or DIPEA, DMAP, DMF, rt, overnight, 84–96%; (b) 4-carbamimidamidobenzoyl chloride hydrochloride, pyridine, acetonitrile, or NMP, 50 °C, overnight, 12–75%; (c) 4 M HCl/EtOAc, rt, overnight, or TFA, rt, 2 h, 40%—quant.

Scheme 2 depicts the synthesis of **2a–c** and **4a–c**. Carboxylic acids **7a** and **7e–g** were subjected to condensation reaction with Boc-protected amino acids to afford amides **8b–g**, which were subsequently treated with 4-carbamimidamidobenzoyl chloride to give precursors **9b–g**. Subsequent deprotection with hydrogen chloride or trifluoroacetic acid afforded the target compounds.

**Scheme 3:**
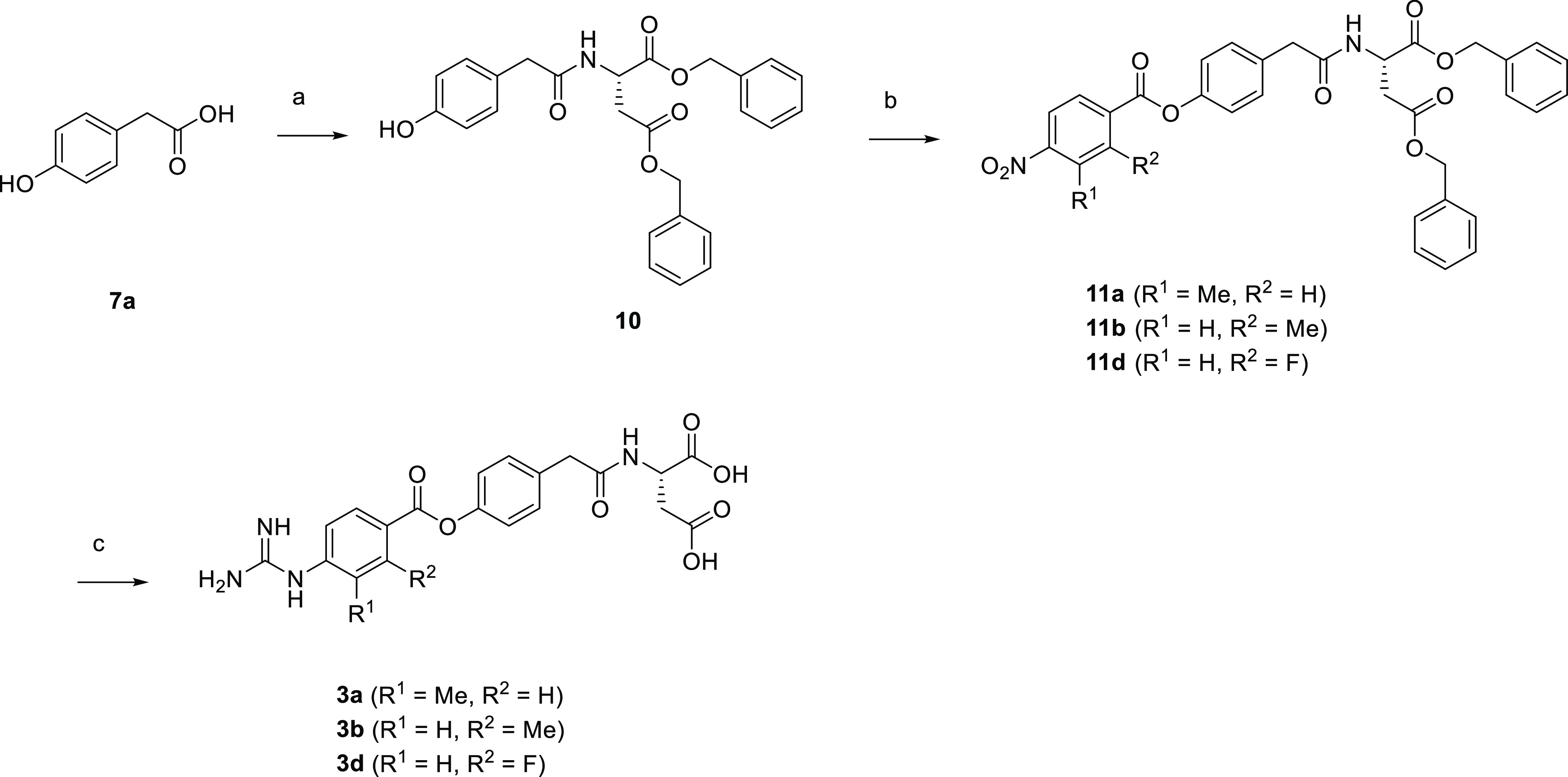
Synthesis of **3a**, **3b**, and **3d** Reagents and conditions: (a) dibenzyl l-aspartate hydrochloride, WSC, HOBt, Et_3_N, DMAP, DMF, rt, overnight, 99%; (b) substituted 4-nitrobenzoic acid, oxalyl chloride, cat. DMF, THF, rt, 15 min to 3 h, then, **10**, pyridine, DMF, rt, overnight, 42–95%; (c) H_2_, Pd/C, THF, rt, 5 h to overnight, then, cyanamide, 4 M HCl/CPME, t-BuOH, 60 °C, 6 h to overnight, 55–60%.

Compound **3a**, **3b**, and **3d** were synthesized as shown in Scheme 3. Amidation reaction of carboxylic acid **7a** with dibenzyl l-aspartate afforded amide **10**, which was subsequently treated with substituted 4-nitrobenzoyl chlorides generated in situ from the corresponding carboxylic acids to give substituted 4-nitrobenzoates **11a**, **11b**, and **11d**. Subsequent hydrogenation followed by guanidination reaction with cyanamide under acidic conditions afforded the target compounds.

**Scheme 4:**
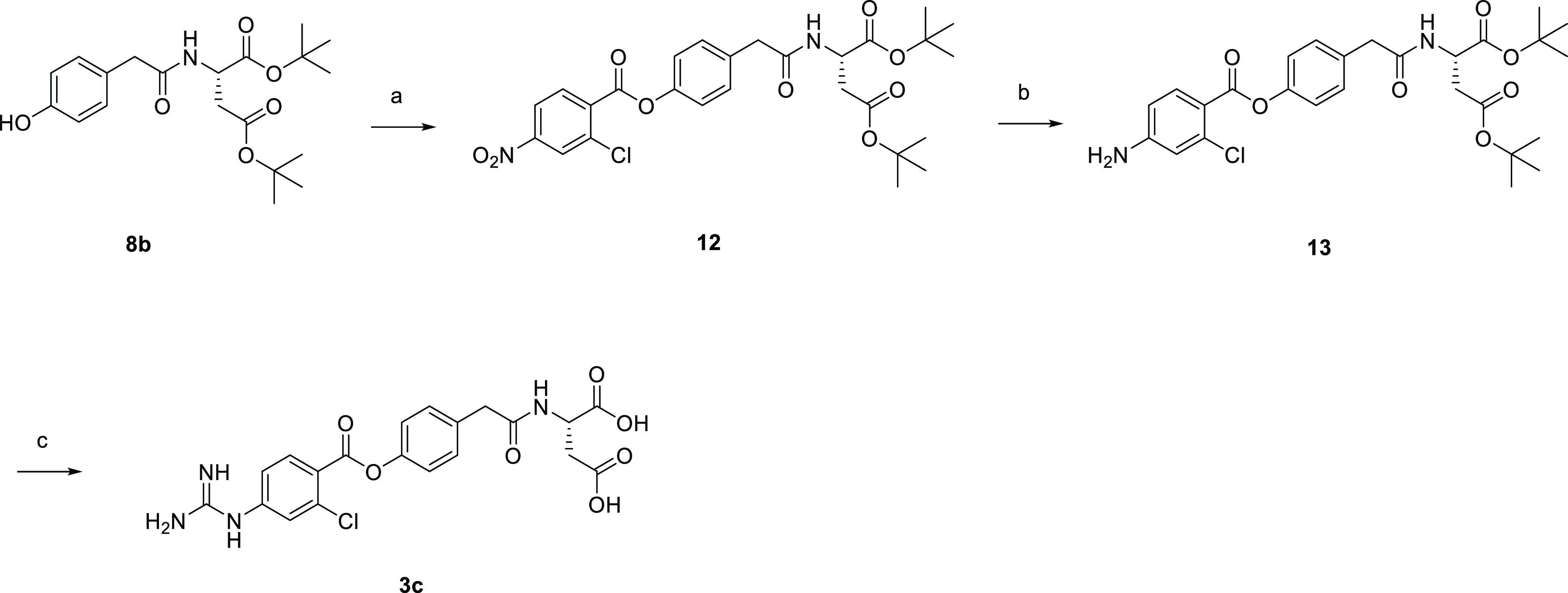
Synthesis of **3c** Reagents and conditions: (a) 2-chloro-4-nitrobenzoyl chloride, pyridine, rt, 5 h, 95%; (b) reduced iron, ammonium chloride, EtOH, water, 75 °C, 1.5 h, 55%; (c) 4 M HCl/CPME, AcOH, rt, overnight, then, cyanamide, 4 M HCl/CPME, *t*-BuOH, 60 °C, 7 h, 49%.

Scheme 4 illustrates the synthesis of **3c**. Phenol **8b** was subjected to esterification reaction with 2-chloro-4-nitrobenzoyl chloride to afford substituted 4-nitrobenzoate **12**, which was subsequently treated with reduced iron to give aniline **13**. Subsequent deprotection with hydrogen chloride followed by guanidination reaction afforded the target compound.

**Scheme 5:**
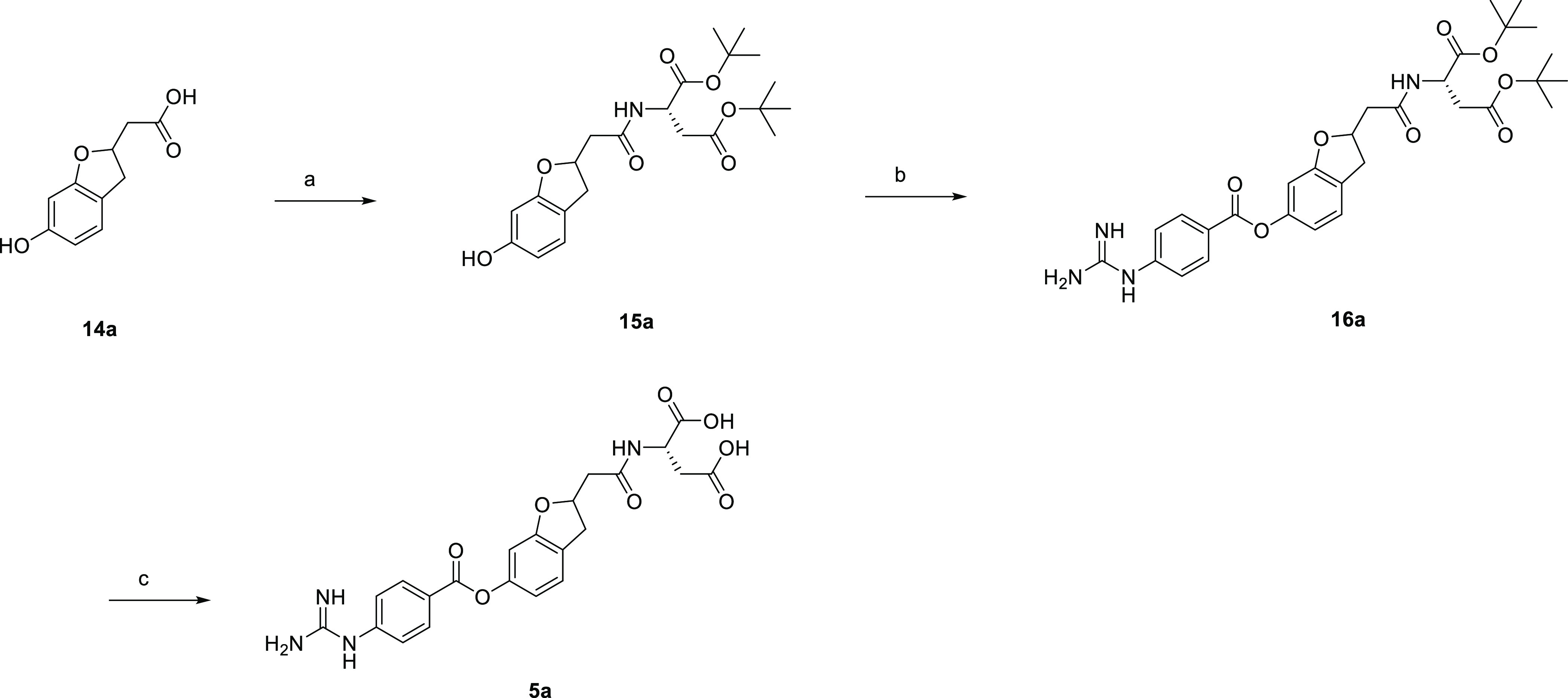
Synthesis of **5a** Reagents and conditions: (a) di-*tert*-butyl l-aspartate hydrochloride, WSC, HOBt, DIPEA, DMF, rt, overnight, 96%; (b) 4-carbamimidamidobenzoyl chloride hydrochloride, pyridine, NMP, 50 °C, overnight, 76%; (c) TFA, rt, 40 min, 60%.

Compound **5a** was synthesized as shown in Scheme 5. Thus, starting from carboxylic acid **14a**, the condensation reaction with di-*tert*-butyl l-aspartate gave amide **15a**, which was converted to guanidyl precursor **16a**. Subsequent deprotection with trifluoroacetic acid afforded **5a**.

**Scheme 6:**
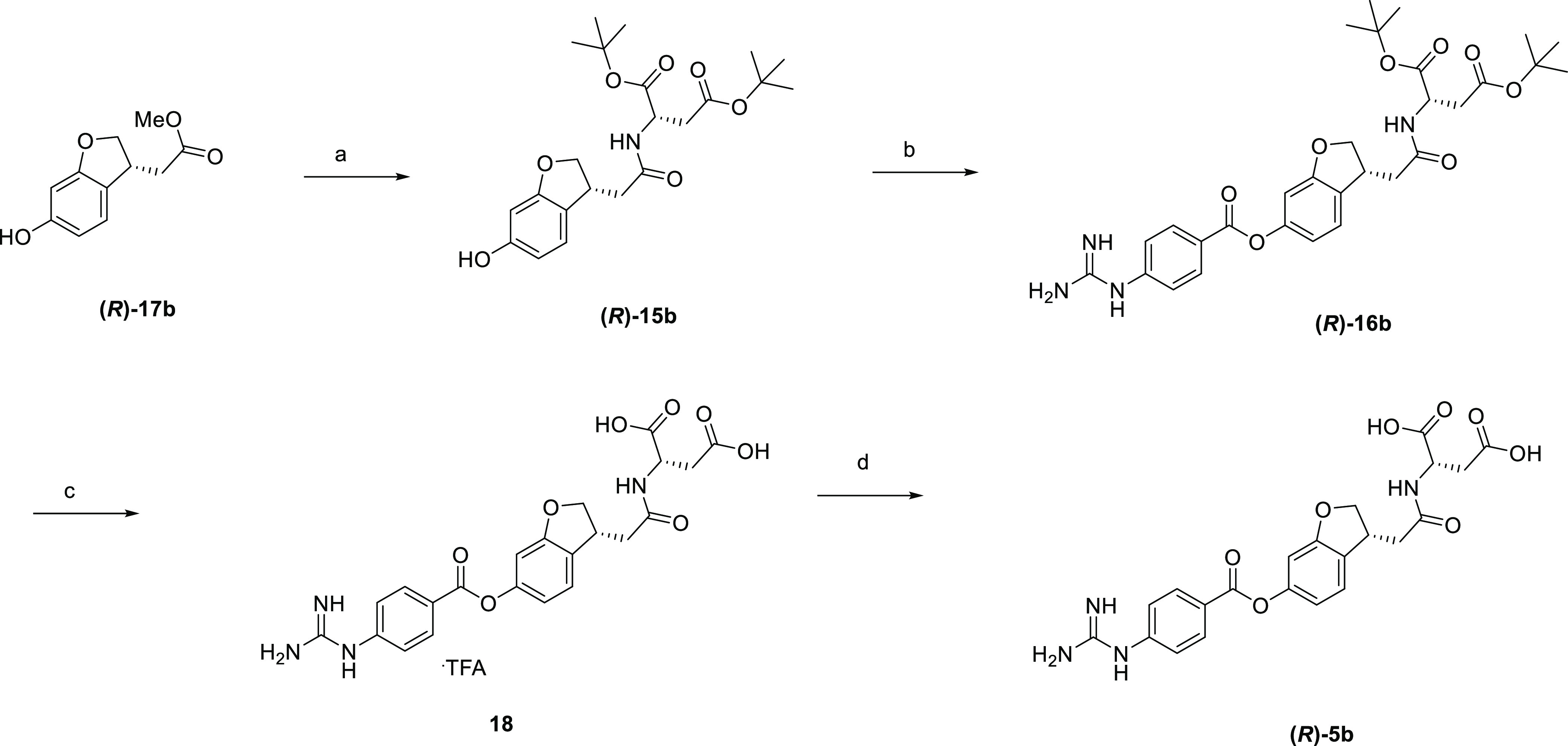
Synthesis of **(*R*)-5b** Reagents and conditions: (a) (1) THF, MeOH, 1 M NaOH, rt, 3 h, (2) di-*tert*-butyl l-aspartate hydrochloride, WSC, HOBt, DIPEA, DMF, rt, overnight, 99%; (b) 4-carbamimidamidobenzoyl chloride hydrochloride, pyridine, acetonitrile, 50 °C, overnight, 64%; (c) TFA, rt, 1 h, 95%; (d) water, acetonitrile, rt, overnight, 91%.

Scheme 6 describes the synthesis of **(*R*)-5b**. Ester **(*R*)-17b**(34) was hydrolyzed to give the corresponding carboxylic acid, which was subjected to condensation reaction with di-*tert*-butyl l-aspartate to afford amide **(*R*)-15b**. Subsequent treatment with 4-carbamimidamidobenzoyl chloride gave precursor **(*R*)-16b**, which was deprotected with trifluoroacetic acid to obtain trifluoroacetic acid salt **18**. Following desalting afforded the target compound in free form.

**Scheme 7:**
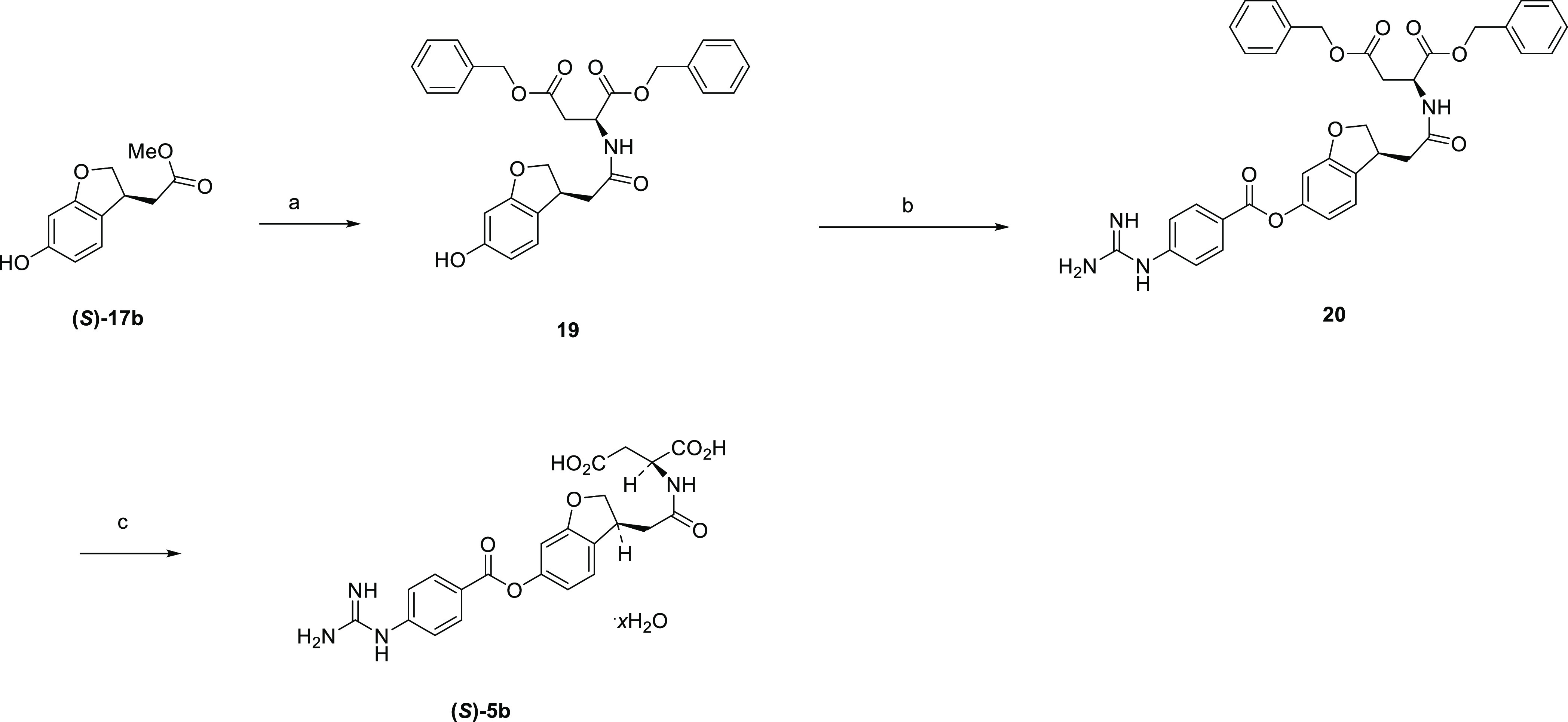
Synthesis of **(*S*)-5b** Reagents and conditions: (a) (1) THF, MeOH, 1 M NaOH, rt, 5 h, (2) dibenzyl l-aspartate hydrochloride, WSC, HOBt, Et_3_N, DIPEA, rt, overnight, 99%; (b) 4-carbamimidamidobenzoyl chloride hydrochloride, pyridine, DMA, 50 °C, overnight; (c) H_2_, Pd/C, 2-propanol, 3 M HCl, rt, 2 h, then water, acetonitrile, rt, overnight, then recrystallization (AcOH–water), 43% from **19**.

Compound **(*S*)-5b** was synthesized as shown in Scheme 7. Hydrolysis of ester **(*S*)-17b**(34) followed by condensation reaction with dibenzyl l-aspartate afforded amide **19**, which was subsequently treated with 4-carbamimidamidobenzoyl chloride to give precursor **20**. Subsequent hydrogenation and desalting followed by recrystallization gave the target compound.

**Figure 11:**
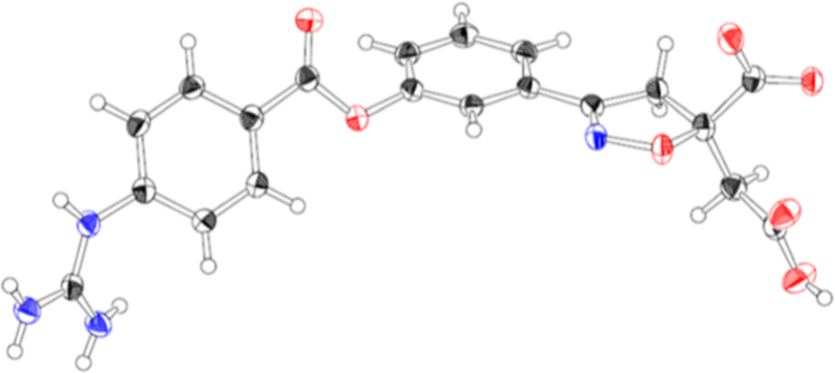
ORTEP of **6b**, thermal ellipsoids are drawn at 50% probability.

**Scheme 8:**
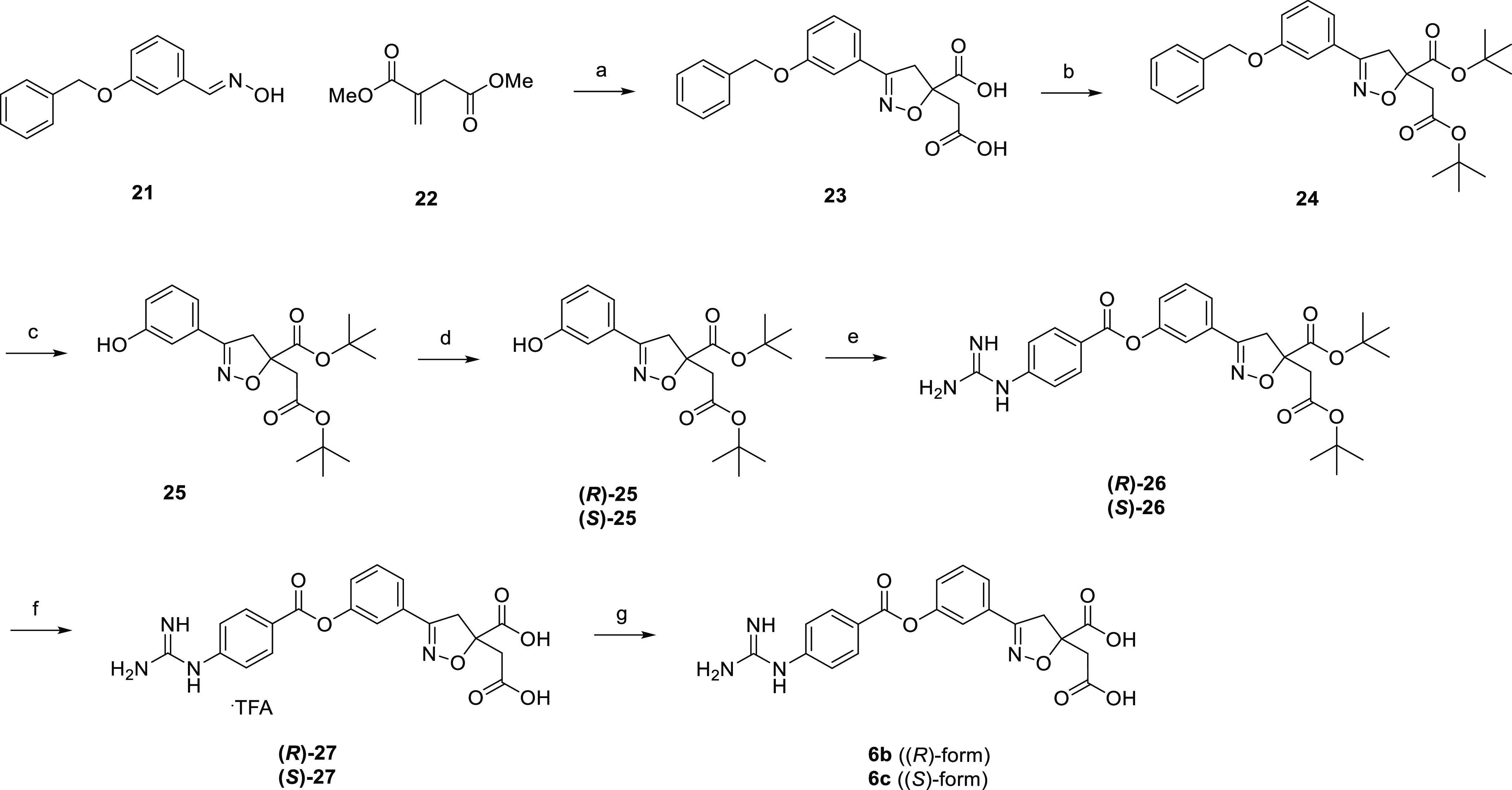
Synthesis of **6b** and **6c** Reagents and conditions: (a) sodium hypochlorite, THF, 0 °C to rt, 1 h, then 2 M NaOH aq., MeOH, 0 °C to rt, overnight, 80%; (b) DMF-di-*tert*-acetal, toluene, 110 °C, 1 h, 83%; (c) H_2_, Pd/C, MeOH, rt, 45 min, 73%; (d) optical resolution, CHIRALPAK AD, 49–50%; (e) 4-carbamimidamidobenzoyl chloride hydrochloride, pyridine, DMA, 50 °C, overnight, 35–56%; (f) TFA, rt, 2 h, 91–96%; (g) water, Et_2_O, 80 °C to rt, overnight, 79–80%.

Scheme 8 illustrates the synthesis of **6b** and **6c**. Aldoxime **21** and dimethyl itaconate **22** were subjected to a [3 + 2] cycloaddition reaction with sodium hypochlorite as an oxidant, followed by hydrolysis to give isoxazoline dicarboxylic acid **23**. Subsequent esterification afforded di-*tert*-butyl ester **24**, which was hydrogenated to give phenol **25**. Optical resolution of **25** by chiral HPLC afforded optical isomers **(*R*)-25** and **(*S*)-25**, which were treated with 4-carbamimidamidobenzoyl chloride to give precursors **(*R*)-26** and **(*S*)-26**. Subsequent deprotection with trifluoroacetic acid gave **(*R*)-27** and **(*S*)-27** as trifluoroacetic acid salts, which were desalted to the target compounds in free form, respectively. The absolute configuration of **6b** was confirmed to be the (*R*)-form by single crystal X-ray structure analysis, whose ORTEP representation is shown in Figure 11.

## Conclusions

Enteropeptidase inhibitors with minimal systemic exposure have been explored to discover potent and safe anti-obesity agents. Based on the docking model of **1c**, we installed an additional carboxylic acid moiety to identify **2a** as a lead compound that display enhanced enteropeptidase inhibitory activity and low membrane permeability. A comparison of the increase in fecal protein output of **2a** between oral and subcutaneous administration revealed that systemic exposure to **2a** was not required for its pharmacological effects. During the course of optimization, non-substituted benzene was identified as the best choice for the LHS benzene ring in terms of IC_50(initial)_ and *T*_1/2_. Furthermore, **4b** showed the most potent enteropeptidase inhibitory activity via rearrangement of the dicarboxylic acid amide moiety to the 3-position of the RHS benzene ring. Considering the unexpectedly weak increase in the fecal protein output after oral administration of **4b** and **4c** in mice, we hypothesized that the stability of the ester and/or amide moieties of a series of analogues might affect the duodenal concentration of the compounds, leading to unexpected results of in vivo efficacy. From **2a**, we designed dihydrobenzofuran analogues, which led to the identification of **(*S*)-5b** (SCO-792) as a potent enteropeptidase inhibitor with extremely low membrane permeability and an enhanced fecal protein output in vivo. In addition, we successfully boosted the increase in the fecal protein output because of **4b** by replacing the amide group with a weaker electron-withdrawing isoxazoline ring (**6b**). Consistent with its extremely low membrane permeability, **(*S*)-5b** and **6b** showed poor plasma exposure in rats, which is consistent with our concept. Furthermore, **(*S*)-5b** and **6b** exhibited potent and durable anti-obesity effects in DIO rats, demonstrating that this novel series of enteropeptidase inhibitors is an attractive candidate for obesity treatment. In addition, the contribution of microbiota to **(*S*)-5b**-induced body weight reduction in DIO mice has been recently reported.^35^ SCO-792 is currently under clinical development.

## Experimental Section

### Chemistry

Melting points were determined on a Yanaco melting point apparatus Mp-500D and are uncorrected. ^1^H NMR and ^13^C NMR spectra were recorded on a Bruker AVANCE III (300 MHz) or a Bruker Advance III plus (400 MHz) spectrometer. Chemical shifts are given in parts per million (ppm) downfield from tetramethylsilane (δ) as the internal standard in the deuterated solvent, and coupling constants (*J*) are in hertz (Hz). Data are reported as follows: chemical shift, integration, multiplicity (s = singlet, d = doublet, t = triplet, q = quartet, quin = quintet, m = multiplet, dd = doublet of doublets, ddd = doublet of doublet of doublets, and br s = broad singlet), and coupling constants. Protons of the dicarboxylic acid group in the 4-guanidinobenzoate analogues (free form) were not observed. Unless otherwise noted, reagents and solvents were obtained from commercial sources and used without further purification. Thin layer chromatography (TLC) was performed on silica gel 60 F254 plates (Merck) or NH TLC plates (Fuji Silysia Chemical Ltd.). Chromatographic purification was performed on Purif-Pack (SI or NH, Fuji Silysia Chemical, Ltd.) or on the UNIVERSAL Column (Silica or Amino, YAMAZEN Corporation). LC–MS analysis was performed on a Shimadzu liquid chromatography–mass spectrometer system, operating in APCI (+ or −) or ESI (+ or −) ionization mode. Analytes were eluted using a linear gradient of 0.05% TFA containing water/acetonitrile or 5 mM ammonium acetate containing the water/acetonitrile mobile phase and detected at 220 nm. Analytical HPLC was performed with a corona charged aerosol detector (CAD). The column was a Capcell Pak C18AQ (50 mm × 3.0 mm I.D., Shiseido, Japan) with a temperature of 50 °C and a flow rate of 0.5 mL/min. Mobile phase A and B were a mixture of 0.2% formic acid in 10 mmol/L ammonium formate and 0.2% formic acid in acetonitrile, respectively. The ratio of mobile phase B was increased linearly from 14 to 86% over 3 min, 86% over the next 1 min. The purities of compounds submitted for biological evaluation were >95% as determined by elemental analyses within ±0.4% of the calculated values or analytical HPLC. Yields are not optimized.

#### *N*-({4-[(4-Carbamimidamidobenzoyl)oxy]phenyl}acetyl)glycine Trifluoroacetic Acid Salt (**1c**)

A mixture of **8a** (210 mg, 0.79 mmol) and 4-carbamimidamidobenzoyl chloride hydrochloride (371 mg, 1.58 mmol) in acetonitrile (1.5 mL) and pyridine (0.3 mL) was stirred at room temperature overnight. The reaction mixture was purified by preparative HPLC (L-column 2, eluted with water in acetonitrile containing 0.1% TFA). The desired fraction was concentrated in vacuo to give 4-{2-[(2-*tert*-butoxy-2-oxoethyl)amino]-2-oxoethyl}phenyl 4-carbamimidamidobenzoate trifluoroacetic acid salt (147 mg) as a yellow gum, which was combined with 4 M HCl/EtOAc (1 mL). After being stirred at room temperature for 4 h, the mixture was concentrated in vacuo. A mixture of the residue and TFA (1 mL) was stirred at room temperature for 1 h. The mixture was concentrated in vacuo, and the solid was collected and washed with EtOAc to give the title compound (114 mg, 30%). ^1^H NMR (400 MHz, DMSO-*d*_6_): δ 3.53 (2H, s), 3.78 (2H, d, *J* = 5.9 Hz), 7.19 (2H, d, *J* = 8.4 Hz), 7.36 (2H, d, *J* = 8.7 Hz), 7.43 (2H, d, *J* = 8.5 Hz), 7.71 (4H, br s), 8.16 (2H, d, *J* = 8.4 Hz), 8.36–8.49 (1H, m), 10.04 (1H, br s), 12.53 (1H, br s). ^13^C NMR (75 MHz, DMSO-*d*_6_): δ 40.7, 41.2, 117.0 (q, *J* = 298.5 Hz), 121.5, 122.8, 125.4, 130.1, 131.4, 134.0, 141.3, 149.1, 155.5, 159.1 (q, *J* = 31.7 Hz), 164.0, 170.4, 171.2. MS (ESI/APCI) *m*/*z*: 371.1 [M + H–TFA]^+^. Anal. Calcd for C_20_H_19_F_3_N_4_O_7_·0.2H_2_O: C, 49.23; H, 4.01; N, 11.48. Found: C, 49.25; H, 4.13; N, 11.19.

#### *N*-({4-[(4-Carbamimidamidobenzoyl)oxy]phenyl}acetyl)-l-aspartic Acid Hydrochloride (**2a**)

A mixture of **9b** (1.70 g, 3.14 mmol) and 4 M HCl/EtOAc (15.7 mL, 62.9 mmol) was stirred at room temperature overnight. The solid was collected to give the title compound (1.46 g, quant.) as a colorless solid. ^1^H NMR (300 MHz, DMSO-*d*_6_): δ 2.56–2.82 (2H, m), 3.53 (2H, s), 4.47–4.61 (1H, m), 7.14–7.23 (2H, m), 7.34 (2H, d, *J* = 8.7 Hz), 7.40–7.49 (2H, m), 7.90 (4H, br s), 8.08–8.21 (2H, m), 8.53 (1H, d, *J* = 7.9 Hz), 10.61 (1H, s), 12.55 (2H, br s). ^13^C NMR (75 MHz, DMSO-*d*_6_): δ 36.5, 41.6, 49.2, 122.0, 123.1, 125.9, 130.6, 131.9, 134.5, 141.6, 149.5, 156.2, 164.5, 170.4, 172.1, 172.8. [α]_D_^20^ – 11.6 (*c* 1.0, DMSO). MS (ESI/APCI) *m*/*z*: 429.1 [M + H–HCl]^+^. Anal. Calcd for C_20_H_21_ClN_4_O_7_·0.6H_2_O: C, 50.50; H, 4.70; N, 11.78. Found: C, 50.47; H, 4.80; N, 11.82.

#### *N*^2^-({4-[(4-Carbamimidamidobenzoyl)oxy]phenyl}acetyl)-l-asparagine Trifluoroacetic Acid Salt (**2b**)

A mixture of **9c** (100 mg, 0.21 mmol) and TFA (2 mL) was stirred at room temperature for 2 h. The mixture was concentrated in vacuo. The residue was washed with Et_2_O/EtOAc. The collected solid was suspended in acetonitrile (3 mL). The suspension was stirred at room temperature overnight. The solid was collected and purified by preparative HPLC (L-column 2, eluted with water in acetonitrile containing 0.1% TFA). The desired fraction was concentrated in vacuo. The residue was washed with Et_2_O to give the title compound (44.4 mg, 40%) as a colorless solid. ^1^H NMR (400 MHz, DMSO-*d*_6_): δ 2.43–2.61 (2H, m), 3.52 (2H, s), 4.44–4.61 (1H, m), 6.90 (1H, br s), 7.18 (2H, d, *J* = 8.6 Hz), 7.31–7.38 (3H, m), 7.43 (2H, d, *J* = 8.7 Hz), 7.74 (4H, br s), 8.16 (2H, d, *J* = 8.7 Hz), 8.35 (1H, d, *J* = 7.9 Hz), 10.12 (1H, br s), 12.54 (1H, br s). ^13^C NMR (101 MHz, DMSO-*d*_6_): δ 36.8, 41.1, 49.0, 117.0 (q, *J* = 299.3 Hz), 121.4, 122.7, 125.4, 130.1, 131.4, 134.1, 141.3, 149.0, 155.5, 158.9 (q, *J* = 31.5 Hz), 164.0, 169.7, 171.2, 172.9. [α]_D_^20^ – 4.6 (*c* 0.25, DMSO). MS (ESI/APCI) *m*/*z*: 428.2 [M + H–TFA]^+^. Anal. Calcd for C_22_H_22_F_3_N_5_O_8_·0.3H_2_O: C, 48.32; H, 4.17; N, 12.81. Found: C, 48.32; H, 4.32; N, 12.90.

#### *N*-({4-[(4-Carbamimidamidobenzoyl)oxy]phenyl}acetyl)-l-glutamic Acid Hydrochloride (**2c**)

The title compound was prepared in quantitative yield using **9d** in an analogous manner to **2a**. Dark yellow amorphous solid. ^1^H NMR (400 MHz, DMSO-*d*_6_): δ 1.72–2.40 (4H, m), 3.43–3.61 (2H, m), 4.12–4.30 (1H, m), 7.19 (2H, d, *J* = 8.3 Hz), 7.32–7.49 (4H, m), 7.77 (4H, br s), 8.16 (2H, d, *J* = 8.5 Hz), 8.43 (1H, d, *J* = 7.8 Hz), 10.26 (1H, s), 11.81–13.02 (2H, m). ^13^C NMR (101 MHz, DMSO-*d*_6_): δ 26.4, 30.1, 41.1, 51.3, 121.5, 122.7, 125.4, 130.1, 131.4, 134.1, 141.1, 149.0, 155.7, 164.0, 170.1, 173.2, 173.6. [α]_D_^20^ – 0.8 (*c* 0.10, DMSO). MS (ESI/APCI) *m*/*z*: 443.1 [M + H–HCl]^+^. Anal. Calcd for C_21_H_23_ClN_4_O_7_·1.5H_2_O: C, 49.86; H, 5.18; N, 11.07. Found: C, 49.73; H, 5.12; N, 11.33.

#### *N*-({4-[(4-Carbamimidamido-3-methylbenzoyl)oxy]phenyl}acetyl)-l-aspartic Acid (**3a**)

Under a H_2_ atmosphere, a mixture of **11a** (880 mg, 1.44 mmol), Pd/C (10% on carbon, wetted with ca. 55% water, 90 mg), and THF (9 mL) was stirred at room temperature for 5 h. The catalyst was filtered off, and the filtrate was concentrated in vacuo. The residue was combined with cyanamide (232 mg, 5.51 mmol) and *t*-BuOH (16 mL). To the mixture was added 4 M HCl/CPME (1.38 mL, 5.51 mmol) at room temperature. The mixture was stirred at 60 °C overnight. The mixture was concentrated in vacuo. To the residue was added water (10 mL), and then, a solution of ammonium acetate (425 mg, 5.51 mmol) in water (2 mL) was added at room temperature dropwise. The mixture was stirred at room temperature for 3 h. The solid was collected and washed with water and acetone to give a crude product (527 mg). 100 mg of the crude was purified by preparative HPLC (L-column 2, eluted with water in acetonitrile containing 0.1% TFA). The desired fraction was concentrated in vacuo. To the residue was added ammonium acetate aq. to make pH ca. 4. The mixture was stirred at room temperature overnight. The solid was collected and washed with water and acetone to give the title compound (68.7 mg, 57%) as a colorless solid. ^1^H NMR (400 MHz, DMSO-*d*_6_): δ 2.25–2.40 (4H, m), 2.46–2.58 (1H, m), 3.44–3.59 (2H, m), 4.18–4.26 (1H, m), 7.12 (2H, d, *J* = 8.6 Hz), 7.25–7.67 (7H, m), 7.95–8.04 (2H, m), 8.10 (1H, d, *J* = 1.6 Hz). ^13^C NMR (101 MHz, DMSO-*d*_6_): δ 17.3, 41.4, 49.0, 121.3, 127.1, 127.4, 128.7, 130.1, 132.4, 134.3, 135.1, 139.3, 148.9, 155.9, 164.1, 169.2, 172.4, 173.5. [α]_D_^20^ + 34.1 (*c* 0.025, DMSO). MS (ESI/APCI) *m*/*z*: 443.1 [M + H]^+^. Anal. Calcd for C_21_H_22_N_4_O_7_·0.7H_2_O: C, 55.43; H, 5.18; N, 12.31. Found: C, 55.48; H, 5.20; N, 12.03.

#### *N*-({4-[(4-Carbamimidamido-2-methylbenzoyl)oxy]phenyl}acetyl)-l-aspartic Acid (**3b**)

The title compound was prepared in 60% yield using **11b** in an analogous manner to **3a** as a colorless solid. ^1^H NMR (400 MHz, DMSO-*d*_6_): δ 2.37 (1H, dd, *J* = 15.7, 4.2 Hz), 2.52–2.60 (4H, m), 3.44–3.59 (2H, m), 4.24 (1H, ddd, *J* = 9.1, 7.4, 4.2 Hz), 7.09 (2H, d, *J* = 8.6 Hz), 7.16–7.25 (2H, m), 7.36 (2H, d, *J* = 8.6 Hz), 7.80 (4H, br s), 7.99 (1H, d, *J* = 7.3 Hz), 8.10 (1H, d, *J* = 8.4 Hz). ^13^C NMR (101 MHz, DMSO-*d*_6_): δ 21.9, 39.6, 42.0, 50.1, 120.8, 122.0, 125.2, 126.1, 130.5, 132.9, 134.8, 141.1, 142.6, 149.4, 156.1, 165.2, 169.7, 173.3, 174.9. [α]_D_^20^ + 42.2 (*c* 0.10, DMSO). MS (ESI/APCI) *m*/*z*: 441.1 [M + H]^+^. Anal. Calcd for C_21_H_22_N_4_O_7_·0.7H_2_O: C, 55.43; H, 5.18; N, 12.31. Found: C, 55.41; H, 4.93; N, 12.19.

#### *N*-({4-[(4-Carbamimidamido-2-chlorobenzoyl)oxy]phenyl}acetyl)-l-aspartic Acid (**3c**)

A mixture of **13** (739 mg, 1.39 mmol), 4 M HCl/CPME (7 mL), and AcOH (7 mL) was stirred at room temperature overnight. The mixture was concentrated in vacuo. The residue was combined with cyanamide (175 mg, 4.17 mmol) and *t*-BuOH (12 mL). To the mixture was added 4 M HCl/CPME (1.04 mL, 4.17 mmol) at room temperature. The mixture was stirred at 60 °C for 7 h. The mixture was concentrated in vacuo. To the residue was added water (10 mL), and then, a solution of ammonium acetate (429 mg, 5.56 mmol) in water (5 mL) was added dropwise. The mixture was stirred at room temperature overnight. The solid was collected and washed with water and acetone to give a crude product (508 mg). 250 mg of the crude was purified by preparative HPLC (L-column 2, eluted with water in acetonitrile containing 0.1% TFA) The desired fraction was concentrated in vacuo. To the residue was added ammonium acetate aq. to make pH ca. 4. The mixture was stirred at room temperature overnight. The solid was collected and washed with water and acetone to give the title compound (156 mg, 49%) as a colorless solid. ^1^H NMR (400 MHz, DMSO-*d*_6_): δ 2.29–2.42 (1H, m), 2.46–2.61 (1H, m), 3.44–3.60 (2H, m), 4.20–4.34 (1H, m), 7.15 (2H, d, *J* = 8.2 Hz), 7.28 (1H, dd, *J* = 8.6, 2.0 Hz), 7.37 (2H, d, *J* = 8.6 Hz), 7.42 (1H, d, *J* = 2.0 Hz), 7.73 (4H, br s), 8.03 (1H, d, *J* = 7.1 Hz), 8.09 (1H, d, *J* = 8.6 Hz). ^13^C NMR (75 MHz, DMSO-*d*_6_): δ 39.4, 42.0, 50.3, 121.7, 121.8, 124.6, 124.9, 130.6, 133.6, 134.4, 135.1, 142.9, 149.2, 156.2, 163.4, 169.8, 173.4, 175.2. [α]_D_^20^ + 11.6 (*c* 0.40, DMSO). MS (ESI/APCI) *m*/*z*: 463.1 [M + H]^+^. Anal. Calcd for C_20_H_19_ClN_4_O_7_·0.4H_2_O: C, 51.10; H, 4.25; N, 11.92. Found: C, 51.04; H, 4.10; N, 11.83.

#### *N*-({4-[(4-Carbamimidamido-2-fluorobenzoyl)oxy]phenyl}acetyl)-l-aspartic Acid (**3d**)

The title compound was prepared in 55% yield using **11d** in an analogous manner to **3a** as a colorless solid. ^1^H NMR (400 MHz, DMSO-*d*_6_): δ 2.38 (1H, dd, *J* = 15.8, 4.1 Hz), 2.51–2.60 (1H, m), 3.44–3.59 (2H, m), 4.20–4.34 (1H, m), 7.10–7.23 (4H, m), 7.33–7.39 (2H, m), 7.71 (4H, br s), 8.00–8.10 (2H, m). ^13^C NMR (101 MHz, DMSO-*d*_6_): δ 39.4, 42.0, 49.8, 111.0 (d, *J* = 24.9 Hz), 113.0 (d, *J* = 9.5 Hz), 118.8, 121.9, 130.6, 133.8, 134.9, 149.2, 155.9, 162.6 (d, *J* = 258.2 Hz), 162.0, 162.1, 169.7, 173.0, 174.5. MS (ESI/APCI) *m*/*z*: 447.1 [M + H]^+^. Anal. Calcd for C_20_H_19_FN_4_O_7_·0.5H_2_O: C, 52.75; H, 4.43; N, 12.30. Found: C, 52.86; H, 4.44; N, 12.33.

#### *N*-({3-[(4-Carbamimidamidobenzoyl)oxy]phenyl}acetyl)-l-aspartic Acid Trifluoroacetic Acid Salt (**4a**)

A mixture of **9e** (248 mg, 0.46 mmol) and TFA (2 mL) was stirred at room temperature for 2 h. The mixture was concentrated in vacuo, and the residue was washed with Et_2_O to give the title compound (190 mg, 76%) as a colorless solid. ^1^H NMR (400 MHz, DMSO-*d*_6_): δ 2.53–2.62 (1H, m), 2.64–2.73 (1H, m), 3.54 (2H, s), 4.44–4.57 (1H, m), 7.08–7.23 (3H, m), 7.33–7.48 (3H, m), 7.78 (4H, br s), 8.09–8.24 (2H, m), 8.44 (1H, d, *J* = 7.7 Hz), 10.19 (1H, br s), 12.74 (2H, br s). ^13^C NMR (75 MHz, DMSO-*d*_6_): δ 36.9, 41.8, 49.3, 117.5 (q, *J* = 298.8 Hz), 120.3, 122.8, 123.2, 125.8, 127.2, 129.7, 131.9, 138.5, 141.9, 150.8, 156.0, 159.6 (q, *J* = 31.4 Hz), 164.4, 170.0, 172.2, 173.0. [α]_D_^20^ – 5.5 (*c* 1.0, DMSO). MS (ESI/APCI) *m*/*z*: 429.1 [M + H–TFA]^+^. Anal. Calcd for C_22_H_21_F_3_N_4_O_9_·0.7H_2_O: C, 47.61; H, 4.07; N, 10.09. Found: C, 47.35; H, 4.45; N, 10.04.

#### *N*-{3-[(4-Carbamimidamidobenzoyl)oxy]benzoyl}-l-aspartic Acid Trifluoroacetic Acid Salt (**4b**)

The title compound was prepared in 77% yield using **9f** in an analogous manner to **4a** as a colorless solid. ^1^H NMR (400 MHz, DMSO-*d*_6_): δ 2.58–2.75 (1H, m), 2.78–2.89 (1H, m), 4.45–4.88 (1H, m), 7.33–7.53 (3H, m), 7.60 (1H, t, *J* = 8.0 Hz), 7.71–7.94 (6H, m), 8.19 (2H, d, *J* = 8.4 Hz), 8.79 (1H, d, *J* = 6.4 Hz), 10.24 (1H, br s), 12.83 (2H, br s). ^13^C NMR (101 MHz, DMSO-*d*_6_): δ 36.2, 49.5, 117.1 (q, *J* = 299.3 Hz), 120.8, 122.7, 125.0, 125.0, 129.7, 131.5, 135.4, 141.6, 150.4, 155.5, 158.7 (q, *J* = 31.5 Hz), 163.9, 164.9, 171.8, 172.6. [α]_D_^20^ – 5.1 (*c* 0.25, DMSO). MS (ESI/APCI) *m*/*z*: 415 [M + H–TFA]^+^. Anal. Calcd for C_21_H_19_F_3_N_4_O_9_·2H_2_O: C, 44.69; H, 4.11; N, 9.93. Found: C, 44.75; H, 4.01; N, 9.66.

#### *N*-{4-[(4-Carbamimidamidobenzoyl)oxy]benzoyl}-l-aspartic Acid Trifluoroacetic Acid Salt (**4c**)

The title compound was prepared in 51% yield using **9g** in an analogous manner to **4a** as a colorless solid. ^1^H NMR (400 MHz, DMSO-*d*_6_): δ 2.62–2.74 (1H, m), 2.79–2.90 (1H, m), 4.66–4.77 (1H, m), 7.40 (2H, d, *J* = 8.7 Hz), 7.44 (2H, d, *J* = 8.7 Hz), 7.78 (4H, br s), 7.97 (2H, d, *J* = 8.7 Hz), 8.18 (2H, d, *J* = 8.7 Hz), 8.74 (1H, d, *J* = 6.7 Hz), 10.22 (1H, br s), 12.83 (2H, br s). MS (ESI/APCI) *m*/*z*: 415.2 [M + H–TFA]^+^. Anal. Calcd for C_21_H_19_F_3_N_4_O_9_·H_2_O: C, 46.16; H, 3.87; N, 10.25. Found: C, 46.27; H, 4.14; N, 10.55.

#### *N*-({6-[(4-Carbamimidamidobenzoyl)oxy]-2,3-dihydro-1-benzofuran-2-yl}acetyl)-l-aspartic Acid (**5a**)

A mixture of **16a** (352 mg, 0.60 mmol) and TFA (3 mL) was stirred at room temperature for 40 min. The mixture was concentrated in vacuo. The residue was purified by preparative HPLC (L-column 2, eluted with water in acetonitrile containing 0.1% TFA). The desired fraction is concentrated in vacuo. To the residue in water (2 mL) was added a solution of ammonium acetate (93.0 mg, 1.21 mmol) in water (1 mL). The mixture was stirred at room temperature. The solid was collected to give the title compound (169 mg, 60%) as a white solid. ^1^H NMR (400 MHz, DMSO-*d*_6_): δ 2.35 (1H, d, *J* = 15.8 Hz), 2.57 (1H, br s), 2.66–2.76 (1H, m), 2.87–3.00 (1H, m), 3.30–3.33 (2H, m), 4.27 (1H, d, *J* = 7.9 Hz), 5.15 (1H, t, *J* = 7.4 Hz), 6.68 (2H, br s), 7.23 (1H, d, *J* = 5.5 Hz), 7.40 (2H, d, *J* = 8.2 Hz), 7.74 (4H, br s), 7.93–8.03 (1H, m), 8.12 (2H, d, *J* = 8.2 Hz). [α]_D_^20^ + 49.0 (*c* 0.040, DMSO). MS (ESI/APCI) *m*/*z*: 471.2 [M + H]^+^. Anal. Calcd for C_22_H_22_N_4_O_8_·H_2_O: C, 54.10; H, 4.95; N, 11.47. Found: C, 53.94; H, 4.89; N, 11.63.

#### *N*-({(3*R*)-6-[(4-Carbamimidamidobenzoyl)oxy]-2,3-dihydro-1-benzofuran-3-yl}acetyl)-l-aspartic Acid (**(*R*)-5b**)

A suspension of **18** (2.90 g, 4.96 mmol) in acetonitrile (15 mL) and water (75 mL) was stirred at room temperature overnight. The solid was collected and washed with acetonitrile/water (1/10 (v/v)) and dried at 60 °C under reduced pressure to give the title compound (2.12 g, 91%) as a white solid. ^1^H NMR (300 MHz, DMSO-*d*_6_): δ 2.23–2.78 (4H, m), 3.71–3.88 (1H, m), 4.30 (2H, dd, *J* = 9.0, 6.6 Hz), 4.69 (1H, t, *J* = 9.1 Hz), 6.65–6.74 (2H, m), 7.26 (1H, d, *J* = 8.6 Hz), 7.40 (2H, d, *J* = 8.6 Hz), 7.70 (4H, br s), 7.99 (1H, d, *J* = 6.6 Hz), 8.13 (2H, d, *J* = 8.6 Hz). [α]_D_^20^ + 50.3 (*c* 0.040, DMSO). MS (ESI/APCI) *m*/*z*: 471.1 [M + H]^+^. Anal. Calcd for C_22_H_22_N_4_O_8_·0.7H_2_O: C, 54.70; H, 4.88; N, 11.60. Found: C, 54.81; H, 4.72; N, 11.41.

#### *N*-({(3*S*)-6-[(4-Carbamimidamidobenzoyl)oxy]-2,3-dihydro-1-benzofuran-3-yl}acetyl)-l-aspartic Acid Hydrate (**(*S*)-5b**)

To a solution of **19** (2.10 g, 4.29 mmol) in DMA (2 mL) and pyridine (2 mL) was added 4-carbamimidamidobenzoyl chloride hydrochloride (2.01 g, 8.58 mmol) at 50 °C. The mixture was stirred at 50 °C overnight. The mixture was purified by column chromatography (silica gel, EtOAc, and then acetonitrile/AcOH = 90/10) to give dibenzyl *N*-({(3*S*)-6-[(4-carbamimidamidobenzoyl)oxy]-2,3-dihydro-1-benzofuran-3-yl}acetyl)-l-aspartate (**20**) (5.04 g, including impurities) as a slightly yellow amorphous solid, which was combined with Pd/C (10% on carbon, wetted with ca. 55% water, 500 mg), 2-propanol (35 mL), and 3 M HCl aq. (7.15 mL, 21.5 mmol). Under a H_2_ atmosphere, the mixture was stirred at room temperature for 2 h. The catalyst was filtered off, and the filtrate was concentrated in vacuo. The residue was dissolved in acetonitrile (10 mL) and water (10 mL). To the solution was added water (40 mL). The mixture was stirred at room temperature overnight. The solid was collected, washed with acetonitrile/water (1/5 (v/v)), and dried under reduced pressure at 60 °C to give the title compound (408 mg) as rough crystals. The filtrate was concentrated in vacuo. The residue was dissolved in a mixture (10 mL) of acetonitrile and water. To the solution was added water (45 mL). The mixture was stirred at room temperature overnight. The solid was collected, washed with acetonitrile/water (1/5 (v/v)), and dried under reduced pressure at 60 °C to give the title compound (740 mg) as rough crystals.

### Recrystallization

To a solution of the rough crystals of the title compound (724 mg) in water (0.5 mL) and AcOH (3.0 mL) was added water (3.5 mL) at 80 °C. The mixture was stirred at 80 °C for 30 min. The mixture was cooled to room temperature. The solid was collected and washed with EtOAc to give the title compound (545 mg, 43% from **19**) as colorless crystals.

^1^H NMR (400 MHz, DMSO-*d*_6_): δ 2.29–2.37 (1H, m), 2.40–2.47 (1H, m), 2.52–2.58 (1H, m), 2.66 (1H, dd, *J* = 14.4, 6.6 Hz), 3.81 (1H, quin, *J* = 7.6 Hz), 4.21–4.32 (2H, m), 4.71 (1H, t, *J* = 9.2 Hz), 6.63–6.70 (2H, m), 7.32 (1H, d, *J* = 7.9 Hz), 7.38 (2H, d, *J* = 8.6 Hz), 7.68 (4H, br s), 7.94 (1H, d, *J* = 7.1 Hz), 8.11 (2H, d, *J* = 8.6 Hz). ^13^C NMR (101 MHz, DMSO-*d*_6_): δ 37.9, 38.8, 40.1, 49.9, 77.2, 103.6, 113.5, 122.4, 124.9, 124.9, 128.1, 131.3, 142.1, 150.7, 155.8, 160.2, 164.0, 169.6, 173.3, 174.8. [α]_D_^20^ + 20.0 (*c* 0.50, DMSO). MS (ESI/APCI) *m*/*z*: 471.2 [M + H]^+^. Anal. Calcd for C_22_H_22_N_4_O_8_·0.4H_2_O: C, 55.32; H, 4.81; N, 11.73. Found: C, 55.36; H, 4.73; N, 11.69. Chiral HPLC analysis (4.6 mm × 250 mm CHIROBIOTIC R column with water/acetonitrile/Et_3_N/AcOH = 900/100/0.3/0.3 at 1.0 mL/min) *t*_R_ = 7.9 min and >99% ee, >99% de.

#### (5*R*)-3-{3-[(4-Carbamimidamidobenzoyl)oxy]phenyl}-5-(carboxymethyl)-4,5-dihydro-1,2-oxazole-5-carboxylic Acid (**6b**)

**(*R*)-27** (7.30 g, 13.5 mmol) was suspended in water (219 mL). The suspension was warmed to 80 °C and sonicated at room temperature, which was repeated several times until insoluble particles were formed. The mixture was cooled to room temperature. To the mixture was added Et_2_O (146 mL), and the mixture was stirred at 0 °C for 2 h and then at room temperature overnight. The mixture was cooled to 0 °C, and the precipitate was collected and washed with water (1 mL) to give the title compound (4.71 g, 79%) as a colorless solid. mp 199 °C (decomposed) (AcOH/water). ^1^H NMR (300 MHz, DMSO-*d*_6_): δ 2.63–2.98 (2H, m), 3.17–3.45 (1H, m), 3.91 (1H, d, *J* = 17.0 Hz), 7.28–7.47 (3H, m), 7.49–7.66 (3H, m), 7.72–8.28 (6H, m). ^13^C NMR (75 MHz, DMSO-*d*_6_): δ 43.6, 44.0, 88.1, 120.4, 122.9, 123.9, 124.6, 125.3, 130.5, 131.5, 132.0, 142.4, 151.2, 156.0, 156.1, 164.4, 171.9, 173.8. [α]_D_^20^ + 131.2 (*c* 1.0, DMSO). MS (ESI/APCI) *m*/*z*: 426.9 [M + H]^+^. Anal. Calcd for C_20_H_18_N_4_O_7_·2H_2_O: C, 51.95; H, 4.80; N, 12.12. Found: C, 52.16; H, 4.78; N, 12.06. Chiral HPLC analysis (4.6 mm × 250 mm CHIROBIOTIC R column with water/acetonitrile/Et_3_N/AcOH = 900/100/1.25/3.75 at 0.5 mL/min) *t*_R_ = 15.3 min and >99.6% ee.

#### (5*S*)-3-{3-[(4-Carbamimidamidobenzoyl)oxy]phenyl}-5-(carboxymethyl)-4,5-dihydro-1,2-oxazole-5-carboxylic Acid (**6c**)

The title compound was prepared in 80% yield using **(*S*)-27** in an analogous manner to **6b** as a colorless solid. ^1^H NMR (400 MHz, DMSO-*d*_6_): δ 2.66–2.97 (2H, m), 3.22–3.38 (1H, m), 3.91 (1H, d, *J* = 17.2 Hz), 7.14–11.06 (12H, m).^13^C NMR (75 MHz, DMSO-*d*_6_): δ 43.4, 44.0, 87.9, 120.4, 122.9, 123.9, 124.7, 125.4, 130.6, 131.4, 132.0, 142.3, 151.2, 156.0, 156.1, 164.4, 171.7, 173.4. [α]_D_^20^ – 119.6 (*c* 0.4, DMSO). MS (ESI/APCI) *m*/*z*: 427.0 [M + H]^+^. Anal. Calcd for C_20_H_18_N_4_O_7_·0.8H_2_O: C, 54.50; H, 4.48; N, 12.71. Found: C, 54.40; H, 4.72; N, 12.60. Chiral HPLC analysis (4.6 mm × 250 mm CHIROBIOTIC R column with water/acetonitrile/Et_3_N/AcOH = 900/100/1/1 at 1.0 mL/min) *t*_R_ = 13.3 min and >99% ee.

#### *tert*-Butyl *N*-[(4-Hydroxyphenyl)acetyl]glycinate (**8a**)

A mixture of **7a** (304 mg, 2.00 mmol), glycine *tert*-butyl ester hydrochloride, (402 mg, 2.40 mmol), WSC (373 mg, 2.40 mmol), HOBt (324 mg, 2.40 mmol), Et_3_N (0.418 mL, 3.00 mmol), and DMF (3 mL) was stirred at room temperature overnight. The mixture was quenched with sat. NaHCO_3_ aq. and extracted with EtOAc. The organic layer was separated, washed with brine, dried over MgSO_4_, and concentrated in vacuo. The residue was purified by column chromatography (silica gel, hexane/EtOAc = 90/10 to 25/75) to give the title compound (508 mg, 96%) as a colorless solid. ^1^H NMR (400 MHz, CDCl_3_): δ 1.44 (9H, s), 3.54 (2H, s), 3.90 (2H, d, *J* = 5.0 Hz), 5.36 (1H, s), 5.90 (1H, br s), 6.81 (2H, d, *J* = 8.3 Hz), 7.14 (2H, d, *J* = 8.2 Hz). MS (ESI/APCI) *m*/*z*: 288.1 [M + Na]^+^.

#### Di-*tert*-butyl *N*-[(4-Hydroxyphenyl)acetyl]-l-aspartate (**8b**)

A mixture of **7a** (5.00 g, 32.9 mmol), di-*tert*-butyl l-aspartate hydrochloride (13.9 g, 49.3 mmol), WSC·HCl (9.45 g, 49.3 mmol), HOBt·H_2_O (7.55 g, 49.3 mmol), DIPEA (17.2 mL, 98.6 mmol), DMAP (401 mg, 3.29 mmol), and DMF (100 mL) was stirred at room temperature overnight. The mixture was quenched with water at room temperature and extracted with EtOAc. The organic layer was separated, washed with water and brine, dried over MgSO_4_, and concentrated in vacuo. The residue was purified by column chromatography (silica gel, hexane/EtOAc = 90/10 to 50/50) to give the title compound (11.9 g, 96%) as a pale yellow amorphous solid. ^1^H NMR (400 MHz, DMSO-*d*_6_): δ 1.35 (9H, s), 1.37 (9H, s), 2.45–2.56 (1H, m), 2.58–2.71 (1H, m), 3.31 (2H, s), 4.33–4.52 (1H, m), 6.66 (2H, d, *J* = 8.2 Hz), 7.03 (2H, d, *J* = 8.2 Hz), 8.23 (1H, d, *J* = 8.2 Hz), 9.20 (1H, s). MS (ESI/APCI) *m*/*z*: 402.1 [M + Na]^+^.

#### *tert*-Butyl *N*^2^-[(4-Hydroxyphenyl)acetyl]-l-asparaginate (**8c**)

A mixture of **7a** (200 mg, 1.31 mmol), *tert*-butyl l-asparaginate (272 mg, 1.45 mmol), WSC (224 mg, 1.45 mmol), HOBt·H_2_O (221 mg, 1.45 mmol), and DMF (5 mL) was stirred at room temperature overnight. Brine was added, and the mixture was extracted with EtOAc/2-propanol (3/1 (v/v)). The organic layer was separated, dried over Na_2_SO_4_, and concentrated in vacuo. The residue was purified by column chromatography (silica gel, hexane/EtOAc = 50/50 to 0/100, and then EtOAc/MeOH = 100/0 to 90/10) to give the title compound (355 mg, 84%) as a colorless oil. ^1^H NMR (400 MHz, DMSO-*d*_6_): δ 1.34 (9H, s), 2.36–2.59 (2H, m), 3.22–3.37 (2H, m), 4.29–4.47 (1H, m), 6.58–6.73 (2H, m), 6.88 (1H, br s), 6.99–7.12 (2H, m), 7.33 (1H, br s), 8.15 (1H, d, *J* = 8.0 Hz), 9.19 (1H, s). MS (ESI/APCI) *m*/*z*: 345.2 [M + Na]^+^.

#### Di-*tert*-butyl *N*-[(4-Hydroxyphenyl)acetyl]-l-glutamate (**8d**)

A mixture of **7a** (1.00 g, 6.57 mmol), di-*tert*-butyl l-glutamate hydrochloride (2.14 g, 7.23 mmol), WSC·HCl (1.51 g, 7.89 mmol), HOBt·H_2_O (1.21 g, 7.89 mmol), Et_3_N (2.20 mL, 15.8 mmol), and DMF (30 mL) was stirred at room temperature overnight. Water was added, and the mixture was extracted with EtOAc. The organic layer was separated, washed with brine, dried over MgSO_4_ and concentrated in vacuo. The residue was purified by column chromatography (silica gel, hexane/EtOAc = 50/50 to 30/70) to give the title compound (2.47 g, 96%) as a colorless oil. ^1^H NMR (300 MHz, DMSO-*d*_6_): δ 1.36 (9H, s), 1.38 (9H, s), 1.62–1.97 (2H, m), 2.14–2.32 (2H, m), 3.31 (2H, s), 4.06–4.19 (1H, m), 6.62–6.71 (2H, m), 6.96–7.09 (2H, m), 8.23 (1H, d, *J* = 7.7 Hz), 9.20 (1H, s). MS (ESI/APCI) *m*/*z*: 416.2 [M + Na]^+^.

#### Di-*tert*-butyl *N*-[(3-Hydroxyphenyl)acetyl]-l-aspartate (**8e**)

The title compound was prepared in 96% yield using **7e** and di-*tert*-butyl l-aspartate hydrochloride in an analogous manner to **8d** as a pale yellow oil. ^1^H NMR (400 MHz, DMSO-*d*_6_): δ 1.35 (9H, s), 1.37 (9H, s), 2.49–2.56 (1H, m), 2.60–2.68 (1H, m), 3.35 (2H, s), 4.36–4.52 (1H, m), 6.58–6.62 (1H, m), 6.64–6.70 (2H, m), 7.00–7.11 (1H, m), 8.29 (1H, d, *J* = 8.1 Hz), 9.25 (1H, s). MS (ESI/APCI) *m*/*z*: 402.2 [M + H]^+^.

#### Di-*tert*-butyl *N*-(3-Hydroxybenzoyl)-l-aspartate (**8f**)

The title compound was prepared in 87% yield using **7f** and di-*tert*-butyl l-aspartate hydrochloride in an analogous manner to **8d** as a colorless oil. ^1^H NMR (400 MHz, DMSO-*d*_6_): d 1.38 (9H, s), 1.40 (9H, s), 2.57–2.68 (1H, m), 2.73–2.90 (1H, m), 4.55–4.75 (1H, m), 6.92 (1H, d, *J* = 7.4 Hz), 7.13–7.37 (3H, m), 8.59 (1H, d, *J* = 8.0 Hz), 9.67 (1H, s). MS (ESI/APCI) *m*/*z*: 388.1 [M + Na]^+^.

#### Di-*tert*-butyl *N*-(4-Hydroxybenzoyl)-l-aspartate (**8g**)

The title compound was prepared in 91% yield using **7g** in an analogous manner to **8b** as a pale yellow amorphous solid. ^1^H NMR (400 MHz, DMSO-*d*_6_): δ 1.22–1.60 (18H, m), 2.63 (1H, dd, *J* = 15.9, 8.1 Hz), 2.72–2.84 (1H, m), 4.51–4.78 (1H, m), 6.80 (2H, d, *J* = 8.7 Hz), 7.70 (2H, d, *J* = 8.7 Hz), 8.20–8.66 (1H, m), 10.00 (1H, s). MS (ESI/APCI) *m*/*z*: 388.2 [M + Na]^+^.

#### Di-*tert*-butyl *N*-({4-[(4-Carbamimidamidobenzoyl)oxy]phenyl}acetyl)-l-aspartate (**9b**)

A mixture of **8b** (3.79 g, 10.0 mmol) and 4-carbamimidamidobenzoyl chloride hydrochloride (3.51 g, 15.0 mmol) in acetonitrile (8 mL) and pyridine (2 mL) was stirred at 50 °C overnight. The reaction mixture was purified by column chromatography (NH silica gel, EtOAc/MeOH = 100/0 to 70/30) to give the title compound (1.70 g, 31%) as a pale yellow solid. ^1^H NMR (400 MHz, DMSO-*d*_6_): δ 1.37 (9H, s), 1.38 (9H, s), 2.52–2.72 (2H, m), 3.49 (2H, s), 4.49 (1H, q, *J* = 6.9 Hz), 5.48 (4H, br s), 6.89 (2H, d, *J* = 7.5 Hz), 7.12 (2H, d, *J* = 8.0 Hz), 7.31 (2H, d, *J* = 8.3 Hz), 7.89 (2H, d, *J* = 8.3 Hz), 8.45 (1H, d, *J* = 8.0 Hz). MS (ESI/APCI) *m*/*z*: 541.4 [M + H]^+^.

#### 4-(2-{[(2*S*)-4-Amino-1-*tert*-butoxy-1,4-dioxobutan-2-yl]amino}-2-oxoethyl)phenyl 4-Carbamimidamidobenzoate (**9c**)

To a mixture of **8c** (1.14 g, 3.54 mmol), pyridine (1 mL), and NMP (1 mL) was added 4-carbamimidamidobenzoyl chloride hydrochloride (828 mg, 3.54 mmol) at room temperature, and the mixture was stirred at 50 °C for 1 h. Then, additional 4-carbamimidamidobenzoyl chloride hydrochloride (828 mg, 3.54 mmol) was further added, and the mixture was stirred at 50 °C overnight. Acetonitrile was added, and the precipitate was filtered off. The filtrate was concentrated in vacuo, and the residue was purified by column chromatography (NH silica gel, hexane/EtOAc = 50/50 to 0/100 and then EtOAc/MeOH = 100/0 to 70/30). The residue was washed with EtOAc to give the title compound (430 mg, 25%) as a colorless solid. ^1^H NMR (400 MHz, DMSO-*d*_6_): δ 1.36 (9H, s), 2.40–2.61 (2H, m), 3.49 (2H, s), 4.36–4.50 (1H, m), 5.48 (4H, br s), 6.89 (3H, br s), 7.08–7.22 (2H, m), 7.28–7.40 (3H, m), 7.85–8.00 (2H, m), 8.34 (1H, d, *J* = 7.8 Hz). MS (ESI/APCI) *m*/*z*: 484.3 [M + H]^+^.

#### Di-*tert*-butyl *N*-({4-[(4-Carbamimidamidobenzoyl)oxy]phenyl}acetyl)-l-glutamate (**9d**)

The title compound was prepared in 75% yield using **8d** in an analogous manner to **9b** as a pale yellow amorphous solid. ^1^H NMR (400 MHz, DMSO-*d*_6_): δ 1.38 (9H, s), 1.39 (9H, s), 1.69–1.97 (2H, m), 2.18–2.37 (2H, m), 3.49 (2H, s), 4.09–4.19 (1H, m), 5.60 (4H, br s), 6.92 (2H, d, *J* = 8.3 Hz), 7.07–7.21 (2H, m), 7.24–7.42 (2H, m), 7.82–8.00 (2H, m), 8.34–8.49 (1H, m). MS (ESI/APCI) *m*/*z*: 555.3 [M + H]^+^.

#### Di-*tert*-butyl *N*-({3-[(4-Carbamimidamidobenzoyl)oxy]phenyl}acetyl)-l-aspartate (**9e**)

To a mixture of **8e** (1.20 g, 3.16 mmol), pyridine (20 mL), and NMP (20 mL) was added 4-carbamimidamidobenzoyl chloride hydrochloride (1.48 g, 6.32 mmol) at room temperature, and the mixture was stirred at 50 °C for 2 h. Then, 4-carbamimidamidobenzoyl chloride hydrochloride (0.740 g, 3.16 mmol) was added, and the mixture was stirred at 50 °C for 2 h. Then, 4-carbamimidamidobenzoyl chloride hydrochloride (0.740 g, 3.16 mmol) was added, and the mixture was stirred at 50 °C overnight. Acetonitrile was added, and the precipitate was filtered off. The filtrate was concentrated in vacuo, and the residue was purified by column chromatography (NH silica gel, hexane/EtOAc = 50/50 to 0/100 and then EtOAc/MeOH = 100/0 to 80/20) and preparative HPLC (L-column 2 ODS, eluted with water in acetonitrile containing 0.1% TFA). The desired fraction was neutralized with sat. NaHCO_3_ aq. and extracted with EtOAc. The organic layer was separated, dried over MgSO_4_, and concentrated in vacuo to give the title compound (248 mg, 15%) as a colorless amorphous powder. ^1^H NMR (400 MHz, DMSO-*d*_6_): δ 1.35 (9H, s), 1.37 (9H, s), 2.51–2.58 (1H, m), 2.63–2.70 (1H, m), 3.53 (2H, s), 4.44–4.52 (1H, m), 7.10–7.23 (3H, m), 7.36–7.43 (3H, m), 7.61 (4H, br s), 8.13 (2H, d, *J* = 8.7 Hz), 8.49 (1H, d, *J* = 8.1 Hz). MS (ESI/APCI) *m*/*z*: 541.3 [M + H]^+^.

#### Di-*tert*-butyl *N*-{3-[(4-Carbamimidamidobenzoyl)oxy]benzoyl}-l-aspartate (**9f**)

The title compound was prepared in 23% yield using **8f** in an analogous manner to **9e** as a pale yellow oil. ^1^H NMR (300 MHz, DMSO-*d*_6_): δ 1.38 (9H, s), 1.40 (9H, s), 2.60–2.72 (1H, m), 2.76–2.88 (1H, m), 4.66–4.78 (1H, m), 7.10–8.33 (12H, m), 8.87 (1H, d, *J* = 7.9 Hz). MS (ESI/APCI) *m*/*z*: 527.3 [M + H]^+^.

#### Di-*tert*-butyl *N*-{4-[(4-Carbamimidamidobenzoyl)oxy]benzoyl}-l-aspartate (**9g**)

The title compound was prepared in 12% yield using **8g** in an analogous manner to **9e** as a light brown oil. ^1^H NMR (300 MHz, CDCl_3_): δ 1.45 (9H, s), 1.48 (9H, s), 2.79–2.90 (1H, m), 2.93–3.04 (1H, m), 4.79–4.94 (1H, m), 6.89–7.79 (9H, m), 7.87 (2H, d, *J* = 8.7 Hz), 8.19 (2H, d, *J* = 8.6 Hz). MS (ESI/APCI) *m*/*z*: 527.3 [M + H]^+^.

#### Dibenzyl *N*-[(4-Hydroxyphenyl)acetyl]-l-aspartate (**10**)

The title compound was prepared in 99% yield using dibenzyl l-aspartate hydrochloride and Et_3_N in an analogous manner to **8b** as a colorless oil. ^1^H NMR (400 MHz, DMSO-*d*_6_): δ 2.75–2.84 (1H, m), 2.86–2.96 (1H, m), 3.31 (2H, s), 4.68–4.76 (1H, m), 5.06 (2H, s), 5.07 (2H, s), 6.64 (2H, d, *J* = 8.4 Hz), 7.00 (2H, d, *J* = 8.4 Hz), 7.25–7.42 (10H, m), 8.51 (1H, d, *J* = 7.9 Hz), 9.19 (1H, s). MS (ESI/APCI) *m*/*z*: 448.2 [M + H]^+^.

#### Dibenzyl *N*-({4-[(3-Methyl-4-nitrobenzoyl)oxy]phenyl}acetyl)-l-aspartate (**11a**)

DMF (3 drops) was added to a mixture of 3-methyl-4-nitrobenzoic acid (486 mg, 2.68 mmol), oxalyl chloride (0.352 mL, 4.02 mmol), and THF (5 mL) at room temperature, and the mixture was stirred at room temperature for 3 h. To the mixture was added a solution of **10** (1.00 g, 2.23 mmol) in DMF (4 mL) and pyridine (2 mL) at 0 °C dropwise. The mixture was stirred at room temperature overnight. 1 M HCl aq. was added at 0 °C, and the mixture was extracted with EtOAc. The organic layer was separated, washed with 0.28% NH_3_ aq. twice, 1 M HCl aq., and brine, dried over MgSO_4_, and concentrated in vacuo. The residue was washed with ^*i*^Pr_2_O to give the title compound (880 mg, 65%) as a colorless solid. ^1^H NMR (400 MHz, DMSO-*d*_6_): δ 2.60 (3H, s), 2.78–2.88 (1H, m), 2.89–2.98 (1H, m), 3.45–3.56 (2H, m), 4.69–4.80 (1H, m), 5.08 (2H, s), 5.09 (2H, s), 7.17–7.25 (2H, m), 7.28–7.39 (12H, m), 8.15 (2H, d, *J* = 0.7 Hz), 8.24 (1H, s), 8.72 (1H, d, *J* = 7.8 Hz). MS (ESI/APCI) *m*/*z*: 611.2 [M + H]^+^.

#### Dibenzyl *N*-({4-[(2-Methyl-4-nitrobenzoyl)oxy]phenyl}acetyl)-l-aspartate (**11b**)

The title compound was prepared in 95% yield using 2-methyl-4-nitrobenzoic acid in an analogous manner to **11a** as a pale yellow solid. ^1^H NMR (400 MHz, DMSO-*d*_6_): δ 2.70 (3H, s), 2.77–2.86 (1H, m), 2.88–2.99 (1H, m), 3.45–3.60 (2H, m), 4.70–4.83 (1H, m), 5.08 (2H, s), 5.09 (2H, s), 7.22 (2H, d, *J* = 8.6 Hz), 7.28–7.46 (12H, m), 8.17–8.31 (3H, m), 8.71 (1H, d, *J* = 7.9 Hz). MS (ESI/APCI) *m*/*z*: 611.2 [M + H]^+^.

#### Dibenzyl *N*-({4-[(2-Fluoro-4-nitrobenzoyl)oxy]phenyl}acetyl)-l-aspartate (**11d**)

DMF (1 drop) was added to a mixture of 2-fluoro-4-nitrobenzoic acid (290 mg, 1.56 mmol), oxalyl chloride (0.205 mL, 2.35 mmol), and THF (3 mL) at room temperature, and the mixture was stirred at room temperature for 15 min. The mixture was concentrated in vacuo. To the residue was added a solution of **10** (700 mg, 1.56 mmol) in pyridine (7 mL) at 0 °C. The mixture was stirred at 0 °C for 2 h and at room temperature overnight. 1 M HCl aq. was added at 0 °C, and the mixture was extracted with EtOAc. The organic layer was separated, washed with 0.28% NH_3_ aq., 1 M HCl aq., and brine, dried over MgSO_4_, and concentrated in vacuo. The residue was purified by preparative HPLC (L-column 2 ODS and eluted with water in acetonitrile containing 0.1% TFA). The desired fraction was neutralized with sat. NaHCO_3_ aq. and extracted with EtOAc. The organic layer was separated, dried over MgSO_4_, and concentrated in vacuo to give the title compound (408 mg, 42%) as a colorless oil. ^1^H NMR (400 MHz, DMSO-*d*_6_): δ 2.78–2.87 (1H, m), 2.89–2.98 (1H, m), 3.46–3.58 (2H, m), 4.68–4.82 (1H, m), 5.08 (2H, s), 5.09 (2H, s), 7.22 (2H, d, *J* = 8.3 Hz), 7.28–7.41 (12H, m), 8.15–8.27 (1H, m), 8.29–8.40 (2H, m), 8.73 (1H, d, *J* = 7.8 Hz). MS (ESI/APCI) *m*/*z*: 615.1 [M + H]^+^.

#### Di-*tert*-butyl *N*-({4-[(2-Chloro-4-nitrobenzoyl)oxy]phenyl}acetyl)-l-aspartate (**12**)

To a solution of **8b** (1.00 g, 2.64 mmol) in pyridine (10 mL) was added 2-chloro-4-nitrobenzoyl chloride (638 mg, 2.90 mmol) at 0 °C. The mixture was stirred at room temperature for 5 h. 1 M HCl aq. was added at 0 °C, and the mixture was extracted with EtOAc. The organic layer was separated, washed with 0.28% NH_3_ aq., 1 M HCl aq., and brine, dried over MgSO_4_, and concentrated in vacuo to give the title compound (1.41 g, 95%) as a pale yellow gum. ^1^H NMR (400 MHz, DMSO-*d*_6_): δ 1.37 (9H, s), 1.38 (9H, s), 2.51–2.60 (1H, m), 2.63–2.72 (1H, m), 3.52 (2H, s), 4.41–4.54 (1H, m), 7.28 (2H, d, *J* = 8.6 Hz), 7.34–7.45 (2H, m), 8.28–8.39 (2H, m), 8.44–8.51 (2H, m). MS (ESI/APCI) *m*/*z*: 585.1 [M + Na]^+^.

#### Di-*tert*-butyl *N*-({4-[(4-Amino-2-chlorobenzoyl)oxy]phenyl}acetyl)-l-aspartate (**13**)

A mixture of **12** (1.41 g, 2.50 mmol), reduced iron (699 mg, 12.5 mmol), ammonium chloride (134 mg, 2.50 mmol), EtOH (12 mL), and water (3 mL) was stirred at 75 °C for 1.5 h. The precipitate was filtered off, and the mixture was extracted with EtOAc. The organic layer was separated, washed with brine, dried over MgSO_4_, and concentrated in vacuo. The residue was purified by column chromatography (silica gel, hexane/EtOAc = 60/40 to 40/60) to give the title compound (739 mg, 55%) as a light brown gum. ^1^H NMR (400 MHz, DMSO-*d*_6_): δ 1.37 (9H, s), 1.38 (9H, s), 2.51–2.58 (1H, m), 2.62–2.74 (1H, m), 3.49 (2H, s), 4.43–4.54 (1H, m), 6.35 (2H, s), 6.58 (1H, dd, *J* = 8.7, 2.2 Hz), 6.70 (1H, d, *J* = 2.2 Hz), 7.07–7.14 (2H, m), 7.25–7.34 (2H, m), 7.87 (1H, d, *J* = 8.7 Hz), 8.43 (1H, d, *J* = 8.1 Hz). MS (ESI/APCI) *m*/*z*: 555.2 [M + Na]^+^.

#### Di-*tert*-butyl *N*-[(6-Hydroxy-2,3-dihydro-1-benzofuran-2-yl)acetyl]-l-aspartate (**15a**)

The title compound was prepared in 96% yield using **14a** and DIPEA in an analogous manner to **8d** as a white amorphous solid. ^1^H NMR (400 MHz, DMSO-*d*_6_): δ 1.40 (18H, s), 2.52–2.80 (5H, m), 3.09–3.20 (1H, m), 4.44–4.54 (1H, m), 5.01 (1H, quin, *J* = 7.5 Hz), 6.12 (1H, br s), 6.21 (1H, d, *J* = 8.3 Hz), 6.88–6.97 (1H, m), 8.35 (1H, d, *J* = 6.8 Hz), 9.16–9.23 (1H, m). MS (ESI/APCI) *m*/*z*: 444.2 [M + Na]^+^.

#### Di-*tert*-butyl *N*-{[(3*R*)-6-Hydroxy-2,3-dihydro-1-benzofuran-3-yl]acetyl}-l-aspartate (**(*R*)-15b**)

To a solution of **(*R*)-17b** (2.55 g, 12.3 mmol) in MeOH (25 mL) and THF (25 mL) was added 1 M NaOH (25.0 mL, 25.0 mmol) at 0 °C. The mixture was stirred at room temperature for 3 h. The mixture was acidified with 1 M HCl aq. (50 mL) at 0 °C and extracted with EtOAc. The organic layer was separated, washed with water and brine, dried over Na_2_SO_4_, and concentrated in vacuo. To a solution of the residue in DMF (100 mL) were added di-*tert*-butyl l-aspartate hydrochloride (5.18 g, 18.4 mmol), DIPEA (6.42 mL, 36.7 mmol), WSC·HCl (3.52 g, 18.4 mmol), and HOBt^.^H_2_O (2.81 g, 18.4 mmol) at 0 °C. The mixture was stirred at room temperature overnight. The reaction was quenched with sat. NH_4_Cl aq. and extracted with EtOAc. The organic layer was separated, washed with 1 M HCl, water, sat. NaHCO_3_ aq., and brine, dried over Na_2_SO_4_, and concentrated in vacuo. The residue was purified by column chromatography (silica gel, hexane/EtOAc = 95/5 to 50/50) to give the title compound (5.09 g, 99%) as a white amorphous solid. ^1^H NMR (300 MHz, DMSO-*d*_6_): δ 1.40 (18H, s), 2.30 (1H, dd, *J* = 14.6, 8.9 Hz), 2.52–2.59 (2H, m), 2.60–2.72 (1H, m), 3.53–3.72 (1H, m), 4.15 (1H, dd, *J* = 9.1, 6.3 Hz), 4.43–4.62 (2H, m), 6.15 (1H, d, *J* = 2.1 Hz), 6.21 (1H, dd, *J* = 8.0, 2.2 Hz), 6.94 (1H, dd, *J* = 8.0, 0.7 Hz), 8.31 (1H, d, *J* = 8.1 Hz), 9.24 (1H, s). MS (ESI/APCI) *m*/*z*: 444.2 [M + Na]^+^.

#### Di-*tert*-butyl *N*-({6-[(4-Carbamimidamidobenzoyl)oxy]-2,3-dihydro-1-benzofuran-2-yl}acetyl)-l-aspartate (**16a**)

The title compound was prepared in 76% yield using **15a** in an analogous manner to **9c** as an off-white amorphous solid. ^1^H NMR (400 MHz, DMSO-*d*_6_): δ 1.40 (18H, s), 2.52–2.78 (5H, m), 2.86–2.95 (1H, m), 4.42–4.56 (1H, m), 5.08–5.21 (1H, m), 5.55 (4H, br s), 6.54–6.61 (1H, m), 6.64 (1H, d, *J* = 8.0 Hz), 6.91 (2H, d, *J* = 8.3 Hz), 7.22 (1H, t, *J* = 7.5 Hz), 7.87 (2H, d, *J* = 8.4 Hz), 8.40 (1H, d, *J* = 8.5 Hz). MS (ESI/APCI) *m*/*z*: 583.3 [M + H]^+^.

#### Di-*tert*-butyl *N*-({(3*R*)-6-[(4-Carbamimidamidobenzoyl)oxy]-2,3-dihydro-1-benzofuran-3-yl}acetyl)-l-aspartate (**(*R*)-16b**)

The title compound was prepared in 64% yield using **(*R*)-15b** in an analogous manner to **9b** as a pale yellow amorphous solid. ^1^H NMR (400 MHz, DMSO-*d*_6_): δ 1.41 (18H, s), 2.37–2.73 (4H, m), 3.71–3.85 (1H, m), 4.27 (1H, t, *J* = 7.5 Hz), 4.47–4.58 (1H, m), 4.68 (1H, t, *J* = 9.1 Hz), 5.49 (4H, br s), 6.59–6.69 (2H, m), 6.89 (2H, d, *J* = 7.5 Hz), 7.23 (1H, d, *J* = 7.9 Hz), 7.87 (2H, d, *J* = 8.0 Hz), 8.39 (1H, d, *J* = 7.5 Hz). MS (ESI/APCI) *m*/*z*: 583.4 [M + H]^+^.

#### *N*-({(3*R*)-6-[(4-Carbamimidamidobenzoyl)oxy]-2,3-dihydro-1-benzofuran-3-yl}acetyl)-l-aspartic Acid Trifluoroacetic Acid Salt (**18**)

**(*R*)-16b** (2.57 g, 4.40 mmol) was dissolved in TFA (50 mL) at 0 °C. The mixture was stirred at room temperature for 1 h. The reaction mixture was concentrated in vacuo. The residue was dissolved in acetonitrile (10 mL) at room temperature. ^*i*^Pr_2_O (100 mL) was added to the mixture. The precipitated solid was collected and washed with acetonitrile/^*i*^Pr_2_O (1/10 (v/v)) to give the title compound (2.44 g, 95%) as a white solid. ^1^H NMR (300 MHz, DMSO-*d*_6_): δ 2.32–2.83 (4H, m), 3.72–3.89 (1H, m), 4.28 (1H, dd, *J* = 9.0, 6.7 Hz), 4.52–4.64 (1H, m), 4.70 (1H, t, *J* = 9.2 Hz), 6.65–6.75 (2H, m), 7.19–7.32 (1H, m), 7.42 (2H, d, *J* = 8.7 Hz), 7.75 (4H, s), 8.13 (2H, d, *J* = 8.7 Hz), 8.37 (1H, d, *J* = 8.0 Hz), 10.14 (1H, s), 12.57 (2H, br s). MS (ESI/APCI) *m*/*z*: 471.2 [M + H–TFA]^+^.

#### Dibenzyl *N*-{[(3*S*)-6-Hydroxy-2,3-dihydro-1-benzofuran-3-yl]acetyl}-l-aspartate (**19**)

To a solution of **(*S*)-17b** (3.07 g, 14.7 mmol) in MeOH (30 mL) and THF (30 mL) was added 1 M NaOH (30.0 mL, 30.0 mmol) at 0 °C. The mixture was stirred at room temperature for 5 h. The mixture was acidified with 1 M HCl aq. (50 mL) at 0 °C and extracted with EtOAc. The organic layer was separated, washed with water and brine, dried over Na_2_SO_4_, and concentrated in vacuo. To a solution of the residue in DMF (50 mL) were added dibenzyl l-aspartate hydrochloride (5.66 g, 16.2 mmol), DIPEA (6.43 mL, 36.8 mmol), WSC·HCl (3.10 g, 16.2 mmol), and HOBt·H_2_O (2.48 g, 16.2 mmol) at 0 °C. The mixture was stirred at room temperature overnight. The reaction was quenched with sat. NH_4_Cl aq. and extracted with EtOAc. The organic layer was separated, washed with 1 M HCl aq., water, sat. NaHCO_3_ aq., and brine, dried over Na_2_SO_4_, and concentrated in vacuo. The residue was purified by column chromatography (silica gel, hexane/EtOAc = 90/10 to 50/50, and then NH silica gel, hexane/EtOAc = 90/10 to 50/50) to give the title compound (7.10 g, 99%) as a white solid. ^1^H NMR (300 MHz, DMSO-*d*_6_): δ 2.27 (1H, dd, *J* = 14.7, 8.8 Hz), 2.44–2.58 (1H, m), 2.74–2.85 (1H, m), 2.86–2.99 (1H, m), 3.53–3.67 (1H, m), 4.07 (1H, dd, *J* = 9.1, 6.4 Hz), 4.49 (1H, t, *J* = 9.0 Hz), 4.66–4.82 (1H, m), 5.10 (4H, d, *J* = 8.9 Hz), 6.15 (1H, d, *J* = 2.2 Hz), 6.20 (1H, dd, *J* = 8.0, 2.2 Hz), 6.94 (1H, dd, *J* = 8.0, 0.6 Hz), 7.26–7.43 (10H, m), 8.54 (1H, d, *J* = 7.8 Hz), 9.26 (1H, s). MS (ESI/APCI) *m*/*z*: 490.2 [M + H]^+^.

#### 3-[3-(Benzyloxy)phenyl]-5-(carboxymethyl)-4,5-dihydro-1,2-oxazole-5-carboxylic Acid (**23**)

To a solution of **21** (42.7 g, 188 mmol) and **22** (29.7 g, 188 mmol) in THF (500 mL) was added sodium hypochlorite (5% aqueous solution, 308 g, 207 mmol) at 0 °C dropwise, and the mixture was stirred at 0 °C for 10 min and at room temperature for 1 h. The mixture was cooled to 0 °C, and then, MeOH (250 mL) and 2 M NaOH aq. (250 mL) were added. The mixture was stirred at room temperature overnight. The mixture was concentrated to half volume. To the residue were added water and EtOAc, and then, the aqueous layer was acidified with 6 M HCl aq. To the mixture was added brine, and the mixture was extracted with EtOAc. The organic layer was separated, washed with brine, dried over MgSO_4_, and concentrated in vacuo. The residue was washed with Et_2_O (10 mL) and excess hexane to give the title compound (53.2 g, 80%) as a light brown solid. ^1^H NMR (400 MHz, DMSO-*d*_6_): δ 2.99 (2H, s), 3.57 (1H, d, *J* = 17.8 Hz), 3.87 (1H, d, *J* = 17.4 Hz), 5.16 (2H, s), 6.71–7.77 (9H, m), 12.55 (1H, br s), 13.21 (1H, br s). MS (ESI/APCI) *m*/*z*: 356.1 [M + H]^+^.

#### *tert*-Butyl 3-[3-(Benzyloxy)phenyl]-5-(2-*tert*-butoxy-2-oxoethyl)-4,5-dihydro-1,2-oxazole-5-carboxylate (**24**)

To a mixture of **23** (14.8 g, 41.7 mmol) in toluene (150 mL) was added *N*,*N*-dimethylformamide di-*t*-butyl acetal (49.9 mL, 208 mmol) dropwise at 110 °C, and the mixture was refluxed for 30 min. Then, additional *N*,*N*-dimethylformamide di-*t*-butyl acetal (12.5 mL, 52.1 mmol) was added, and the mixture was reflued for 30 min. The mixture was concentrated in vacuo, and the residue was purified by column chromatography (silica gel, hexane/EtOAc = 99/1 to 80/20) to give the title compound (16.2 g, 83%) as a pale yellow oil. ^1^H NMR (400 MHz, DMSO-*d*_6_): δ 1.40 (9H, br s), 1.42 (9H, br s), 2.87–3.08 (2H, m), 3.54 (1H, d, *J* = 17.4 Hz), 3.85 (1H, d, *J* = 17.6 Hz), 5.16 (2H, s), 7.00–7.64 (9H, m). MS (ESI/APCI) *m*/*z*: 490.2 [M + Na]^+^.

#### *tert*-Butyl 5-(2-*tert*-Butoxy-2-oxoethyl)-3-(3-hydroxyphenyl)-4,5-dihydro-1,2-oxazole-5-carboxylate (**25**)

Under a H_2_ atmosphere, a mixture of **24** (58.4 g, 125 mmol), Pd/C (10% on carbon, wetted with ca.55% water, 5.80 g), and MeOH (500 mL) was stirred at room temperature for 45 min. The catalyst was filtered off, and the filtrate was concentrated in vacuo. The residue was purified by column chromatography (silica gel, hexane/EtOAc = 99/1 to 50/50) to give the title compound (34.5 g, 73%) as a pale yellow solid. ^1^H NMR (400 MHz, DMSO-*d*_6_): δ 1.40 (9H, br s), 1.42 (9H, br s), 2.84–3.08 (2H, m), 3.52 (1H, d, *J* = 17.6 Hz), 3.79 (1H, d, *J* = 17.7 Hz), 6.87 (1H, d, *J* = 8.2 Hz), 7.03–7.15 (2H, m), 7.19–7.34 (1H, m), 9.66 (1H, s). MS (ESI/APCI) *m*/*z*: 400.2 [M + Na]^+^.

#### *tert*-Butyl (5*R*)-5-(2-*tert*-Butoxy-2-oxoethyl)-3-(3-hydroxyphenyl)-4,5-dihydro-1,2-oxazole-5-carboxylate (**(*R*)-25**) and *tert*-Butyl (5*S*)-5-(2-*tert*-Butoxy-2-oxoethyl)-3-(3-hydroxyphenyl)-4,5-dihydro-1,2-oxazole-5-carboxylate (**(*S*)-25**)

**25** (49.5 g, 131 mmol) was purified by chiral preparative HPLC (50 mm × 500 mm CHIRALPAK AD column with hexane/EtOH = 850/150). The first eluting fraction was collected and concentrated in vacuo to give **(*R*)-25** (24.7 g, 50%) as a pale yellow oil. ^1^H NMR (400 MHz, DMSO-*d*_6_): δ 1.40 (9H, s), 1.42 (9H, s), 2.78–3.08 (2H, m), 3.52 (1H, d, *J* = 17.4 Hz), 3.79 (1H, d, *J* = 17.4 Hz), 6.77–6.89 (1H, m), 6.98–7.15 (2H, m), 7.16–7.34 (1H, m), 9.66 (1H, s). *m*/*z*: 400.2 [M + Na]^+^. Chiral HPLC analysis (4.6 mm × 250 mm CHIRALPAK AD column with hexane/EtOH = 850/150 at 1.0 mL/min) *t*_R_ = 6.07 min and >99.9% ee. The second eluting fraction was collected and concentrated in vacuo to give **(*S*)-25** (24.3 g, 49%) as a pale yellow oil. ^1^H NMR (400 MHz, DMSO-*d*_6_): δ 1.40 (9H, s), 1.42 (9H, s), 2.82–3.13 (2H, m), 3.52 (1H, d, *J* = 17.4 Hz), 3.79 (1H, d, *J* = 17.4 Hz), 6.72–6.91 (1H, m), 6.98–7.15 (2H, m), 7.18–7.36 (1H, m), 9.66 (1H, br s). MS (ESI/APCI) *m*/*z*: 400.2 [M + Na]^+^. Chiral HPLC analysis (4.6 mm × 250 mm CHIRALPAK AD column with hexane/EtOH = 850/150 at 1.0 mL/min) *t*_R_ = 11.5 min and >99.9% ee.

#### *tert*-Butyl (5*R*)-5-(2-*tert*-Butoxy-2-oxoethyl)-3-{3-[(4-carbamimidamidobenzoyl)oxy]phenyl}-4,5-dihydro-1,2-oxazole-5-carboxylate (**(*R*)-26**)

To a mixture of **(*R*)-25** (15.0 g, 39.7 mmol), pyridine (20 mL), and DMA (20 mL) was added 4-carbamimidamidobenzoyl chloride hydrochloride (23.3 g, 99.4 mmol) at 50 °C. The mixture was stirred at 50 °C overnight. Then, additional 4-carbamimidamidobenzoyl chloride hydrochloride (23.3 g, 99.4 mmol) was added, and the mixture was stirred at 50 °C for 4 h. The mixture was poured into acetonitrile dropwise with stirring at room temperature. The precipitate was filtered off, and then, the filtrate was concentrated in vacuo. The residue was purified by column chromatography (silica gel, EtOAc, and then acetonitrile/AcOH = 90/10). The desired fraction was collected and concentrated in vacuo. The residue was diluted with EtOAc, and the mixture was poured into sat.NaHCO_3_ aq. and extracted with EtOAc. The organic layer was separated, dried over MgSO_4_, and concentrated in vacuo to give the title compound (7.55 g, 35%) as a colorless gum. ^1^H NMR (400 MHz, DMSO-*d*_6_): δ 1.40 (9H, s), 1.42 (9H, s), 2.83–3.09 (2H, m), 3.58 (1H, d, *J* = 17.7 Hz), 3.89 (1H, d, *J* = 17.6 Hz), 6.94–8.45 (12H, m). MS (ESI/APCI) *m*/*z*: 539.3 [M + H]^+^.

#### *tert*-Butyl (5*S*)-5-(2-*tert*-Butoxy-2-oxoethyl)-3-{3-[(4-carbamimidamidobenzoyl)oxy]phenyl}-4,5-dihydro-1,2-oxazole-5-carboxylate (**(*S*)-26**)

The title compound was prepared in 56% yield using **(*S*)-25** in an analogous manner to **(*R*)-26** as a colorless gum. ^1^H NMR (400 MHz, DMSO-*d*_6_): δ 1.40 (9H, s), 1.42 (9H, s), 2.87–3.07 (2H, m), 3.58 (1H, d, *J* = 17.7 Hz), 3.89 (1H, d, *J* = 17.6 Hz), 6.79–8.33 (12H, m). MS (ESI/APCI) *m*/*z*: 539.3 [M + H]^+^.

#### (5*R*)-3-{3-[(4-Carbamimidamidobenzoyl)oxy]phenyl}-5-(carboxymethyl)-4,5-dihydro-1,2-oxazole-5-carboxylic Acid Trifluoroacetic Acid Salt (**(*R*)-27**)

The title compound was prepared in 96% yield using **(*R*)-26** in an analogous manner to **4a** as a colorless solid. ^1^H NMR (400 MHz, DMSO-*d*_6_): δ 3.02 (2H, s), 3.61 (1H, d, *J* = 17.6 Hz), 3.90 (1H, d, *J* = 17.6 Hz), 7.36–7.50 (3H, m), 7.54–7.70 (3H, m), 7.78 (4H, br s), 8.18 (2H, d, *J* = 8.7 Hz), 10.17 (1H, br s), 12.88 (2H, br s). MS (ESI/APCI) *m*/*z*: 427.1 [M + H–TFA]^+^.

#### (5*S*)-3-{3-[(4-Carbamimidamidobenzoyl)oxy]phenyl}-5-(carboxymethyl)-4,5-dihydro-1,2-oxazole-5-carboxylic Acid Trifluoroacetic Acid Salt (**(*S*)-27**)

The title compound was prepared in 91% yield using **(*S*)-26** in an analogous manner to **4a** as a colorless solid. ^1^H NMR (400 MHz, DMSO-*d*_6_): δ 3.02 (2H, s), 3.61 (1H, d, *J* = 17.6 Hz), 3.90 (1H, d, *J* = 17.6 Hz), 7.37–7.50 (3H, m), 7.53–7.67 (3H, m), 7.80 (4H, br s), 8.18 (2H, d, *J* = 8.7 Hz), 10.21 (1H, br s), 12.62 (s, 1 H) 13.01 (s, 1 H). MS (ESI/APCI) *m*/*z*: 427.1 [M + H–TFA]^+^.

### Human Enteropeptidase Enzyme Assay

Human recombinant enteropeptidase (#REN-260, ITSI-Biosciences, LLC) was diluted with an assay buffer (50 mM tricine (pH 8.0), 0.01(w/v)% Tween 20, 10 mM CaCl_2_) to prepare a 24 mU/mL enzyme solution. Subsequently, 5FAM–Abu–Gly–Asp–Asp–Asp–Lys–Ile–Val–Gly–Gly–Lys(CPQ2)–Lys–Lys–NH_2_ (purity: 97.2%, CPC Scientific, Inc.) was diluted with an assay buffer to prepare a 2.1 μM substrate solution. Compounds were dissolved in DMSO and then diluted in the assay buffer. The compound solution (5 μL/well) and the substrate solution (5 μL/well) were added to a 384-well black plate (#784076, Greiner Bio-One) and mixed. Then, the enzyme solution (5 μL/well) was added to the plate and mixed to start the reaction. The fluorescence intensity was measured at an excitation wavelength of 485 nm and a fluorescence wavelength of 535 nm using a fluorescence plate reader EnVision (PerkinElmer Inc.). Also, the same reaction as above was performed except that the test compound was not added (test compound non-supplemented group). In addition, the same reaction as above was performed except that neither the test compound nor the enzyme was added (control group). The inhibition rate was calculated from the fluorescence intensity 6 or 120 min after the start of the reaction according to the following equation:

Inhibition rate (%) = (1 – (fluorescence intensity of the test compound supplemented group – fluorescence intensity of the control group)/(fluorescence intensity of the test compound non-supplemented group – fluorescence intensity of the control group)) × 100.

### Rat Enteropeptidase Enzyme Assay

The rat enteropeptidase catalytic subunit (aa 818-1057, GenBank accession no. XM_017597944) was cloned into the BglII-NotI site of the pET32a vector (Millipore). The active rat enteropeptidase catalytic subunit was obtained as described previously.^36^ The rat recombinant enteropeptidase was diluted with an assay buffer (50 mM Tricine (pH 8.0), 0.01(w/v)% Tween 20, 10 mM CaCl_2_) to prepare a 2.4 ng/mL enzyme solution. Subsequently, 5FAM–Abu–Gly–Asp–Asp–Asp–Lys–Ile–Val–Gly–Gly–Lys(CPQ2)–Lys–Lys–NH_2_ (purity: 97.2%, CPC Scientific, Inc.) was diluted with an assay buffer to prepare a 5.4 μM substrate solution. Compounds were dissolved in DMSO and then diluted in the assay buffer. The compound solution (5 μL/well) and the substrate solution (5 μL/well) were added to a 384-well black plate (#784076, Greiner Bio-One) and mixed. Then, the enzyme solution (5 μL/well) was added to the plate and mixed to start the reaction. The fluorescence intensity was measured at an excitation wavelength of 485 nm and a fluorescence wavelength of 535 nm using a fluorescence plate reader EnVision (PerkinElmer Inc.). Also, the same reaction as above was performed except that the test compound was not added (test compound non-supplemented group). In addition, the same reaction as above was performed except that neither the test compound nor the enzyme was added (control group). The inhibition rate was calculated from the fluorescence intensity 120 min after the start of the reaction according to the following equation:

Inhibition rate (%) = (1 – (fluorescence intensity of the test compound supplemented group – fluorescence intensity of the control group)/(fluorescence intensity of the test compound non-supplemented group – fluorescence intensity of the control group)) × 100.

### Dissociation Assay

For dissociation assay, compounds were dissolved in DMSO and then diluted in assay buffer (50 mM Tricine pH 8.0, 0.01(w/v)% Tween20, 10 mM CaCl_2_). 10 μL of compound solution was added in 96-well plate (corning), and then, 10 μL of 100 mU/mL human recombinant enteropeptidase (ITSI-Biosciences) solution was added into the plate and incubated at room temperature for 2 h. The concentration of the compound was equal to 10-fold the IC_50_ value at 120 min incubation. After incubation, 2 μL of the compound-enzyme mixture was transferred to a 96-well black plate (Corning) and 200 μL of substrate solution (3 μM 5FAM–Abu–Gly–Asp–Asp–Asp–Lys–Ile–Val–Gly–Gly–Lys(CPQ2)–Lys–Lys–NH_2_ (CPC Scientific)) was added into the well. By rapid dilution, the inhibitor concentration went to 10-fold below the IC_50_ from 10-fold above the IC_50_. The fluorescence was measured each 2 min at excitation wavelength 485 nm and emission wavelength 535 nm by EnVision (Perkin Elmer). The progress curves were fitted to following equation to determine the values for *k*_off_ and dissociation half-life *T*_1/2_ according to the following equations.
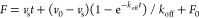
1where *t* is time and *F* is fluorescence from initial rate *v*_o_ to steady state rate *v*_s_.
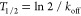
2

### Development of the Docking Model Using the X-ray Crystal Structure

To predict the binding modes of **1c**, **2a**, and **4b**, docking studies were performed using Glide (ver. 72015, Schrödinger, LLC, New York). With the known crystal structure (PDB ID: 4DGJ), the initial structure was prepared using protein preparation wizard with default settings and used as the docking templates. Glide docking of **1c** was performed to obtain 10 docking modes, which were sorted using the Glide Emodel scores. The binding free energies of the top five modes were calculated using the molecular mechanics generalized Born surface area (MM/GBSA) method. The mode with the highest MM/GBSA score was selected as the presumed binding mode. For **2a** and **4b**, induced-fit docking was performed to obtain a maximum of 18 modes, which were sorted by Glide IFD scores. After excluding the modes in which the ester bond differed from the catalytic triad, the binding free energies of each of the top three modes were evaluated with MM/GBSA scores using Prime (ver. 4.5, Schrödinger, LLC). The mode with the best MM/GBSA score was selected as the presumed binding mode.

### X-ray Structure Analysis

Crystal data for **6b**: C_20_H_18_N_4_O_7_·H_2_O, MW = 444.40; crystal size, 0.17 × 0.17 × 0.09 mm; colorless, block; monoclinic, space group *P*2_1_, *a* = 10.8406(2) Å, *b* = 6.04831(11) Å, *c* = 16.0119(12) Å, α = γ = 90°, β = 107.512(8)°, *V* = 1001.20(9) Å^3^, *Z* = 2, Dx = 1.474 g/cm^3^, *T* = 100 K, μ = 0.988 mm^–1^, λ = 1.54187 Å, *R*_1_ = 0.026, w*R*_2_ = 0.071, Flack Parameter^37^ = −0.04(13). All measurements were made on a Rigaku R-AXIS RAPID-191R diffractometer using graphite monochromated Cu Kα radiation. The structure was solved by direct methods with SIR2008^38^ and was refined using full-matrix least-squares on *F*^2^ with SHELXL-97.^39^ All non-H atoms were refined with anisotropic displacement parameters. CCDC 1539971 for compound **6b** contains the supplementary crystallographic data for this paper. These data can be obtained free of charge from The Cambridge Crystallographic Data Centre via https://www.ccdc.cam.ac.uk/structures/.

### Evaluation of Membrane Permeability with Human Multi-drug Resistance 1 (MDR-1) Expressing Cells

The transcellular transport study was performed as reported previously.^40^ In brief, the cells were grown in a HTS transwell 96 well permeable support (pore size 0.4 μm, 0.143 cm^2^ surface area) with a polyethylene terephthalate membrane (Corning Life Sciences, Lowell, MA, USA) at a density of 1.125 × 10^5^ cells/well. The cells were preincubated with M199 at 37 °C for 30 min. Subsequently, transcellular transport was initiated by the addition of M199 to apical compartments containing 10 μmol/L test compounds and terminated by the removal of each assay plate after 2 h. The aliquots in the opposite compartments were subjected to be measured for compound concentration by LC–MS/MS. Permeability was calculated using the permeated compound concentration.

### Measurement of Aqueous Stability at pH 1.2/6.8

Sample solutions in pH 1.2/6.8 media (first/second fluids for the disintegration test in the Japanese Pharmacopoeia 17th edition, including 10% dimethyl sulfoxide if needed, 10 μg/mL) were prepared for the compounds and incubated at 37 °C. The sample solutions were measured initially and at 24 h using HPLC, and peak analysis was conducted. Residual contents (%) of the compounds in pH 1.2/6.8 media at 37 °C after 24 h of incubation of the initial solutions were calculated using the peak areas.

### Animal Experiments

All animal experiments were performed in compliance with the guidelines for the Care and Use of Laboratory Animals of Takeda Pharmaceutical Company Ltd.

### Pharmacokinetic Analysis of Rat Cassette Dosing

The test compound was administered orally (1 mg/kg, suspended in 0.5% methylcellulose aqueous solution) via cassette dosing to non-fasted SD rats. After administration, blood samples were collected and centrifuged to obtain plasma specimens. The compound concentrations in the plasma were measured using LC–MS/MS.

### Evaluation of the Increase in Fecal Protein Output in High-Fat-Diet-Fed Mice

A high-fat diet (HFD)-fed mouse (D12079B diet, male, 14–21 weeks old) was used for the in vivo assay. For compound screening, mice were orally administered with either the test compound (10 mg/kg) or 0.5% methylcellulose (MC) as a vehicle control. For the assessment of efficacy by systemic exposure of the compound, mice were subcutaneously administered with compound **2a** (10 mg/kg) or saline as a vehicle control and compared with those administered oral treatment (60 mg/kg). Doses were selected to achieve equivalent systemic exposure as the area under the curve over 24 h based on pharmacokinetic data of both oral and subcutaneous administration. Whole feces were collected overnight, and the fecal protein output was evaluated as described previously.^33^

### Evaluation of Anti-obesity Effects Using Male DIO Rats

Six-week-old male DIO F344/Jcl rats were obtained from CLEA Japan (Tokyo, Japan) and fed with a HFD D12451 (45 kcal % fat, 20 kcal % protein, and 35 kcal % carbohydrate; Research Diets, Inc.). The study involved six groups of rats (*n* = 6): vehicle (0.5% MC), **6b** (1, 3, and 10 mg/kg), **(*S*)-5b** (SCO-792) (10 mg/kg), and sibutramine (1 mg/kg). Mice were orally administered with either the vehicle or compounds starting at 36 weeks of age for 4 weeks. The body weight was monitored every 1–4 days for 4 weeks. The change in body weight is presented as a percentage of the initial body weight. Feces were collected on day 28 and evaluated for pharmacodynamic markers.

## Supporting Information Available

Coordinate file for the docking model of compound **1c** and enteropeptidase (PDB)
Coordinate file for the docking model of compound **2a** and enteropeptidase (PDB)
Coordinate file for the docking model of compound **4b** and enteropeptidase (PDB)
HPLC traces (PDF)
Molecular formula strings (CSV)

## Supplementary Material

jm2c00463_si_001.pdb

jm2c00463_si_002.pdb

jm2c00463_si_003.pdb

## Abbreviations

CPME: cyclopentyl methyl ether
DIO: diet-induced obese
DIPEA: *N*,*N*-diisopropylethylamine
HFD: high-fat diet
HOBt: 1-hydroxybenzotriazole
LHS: left-hand side
MC: methylcellulose
MDR1: multi-drug resistance 1
PAMPA: parallel artificial membrane permeability assay
RHS: right-hand side
WSC: 1-ethyl-3-(3-(dimethylamino)propyl) carbodiimide
