# Supplementary material for: Design, Synthesis, and Biological Evaluation of a Novel Series of 4-Guanidinobenzoate Derivatives as Enteropeptidase Inhibitors with Low Systemic Exposure for the Treatment of Obesity
Source: J Med Chem. 2022 Jun 10;65(12):8456–77. doi: 10.1021/acs.jmedchem.2c00463 (PMC9234964; doi:10.1021/acs.jmedchem.2c00463)
Supplement: Supplementary file 4 — jm2c00463_si_004.pdf [file jm2c00463_si_004.pdf]

Supporting Information

Design, Synthesis, and Biological Evaluation of a Novel Series of 4-guanidinobenzoate Derivatives as Enteropeptidase Inhibitors with Low Systemic Exposure for the Treatment of Obesity

Zenichi Ikeda,[,†] Keiko Kakegawa,[†] Fumiaki Kikuchi,[†] Sachiko Itono,[†,||] Hideyuki Oki,[†,||] Hiroaki Yashiro,[†] Hideyuki Hiyoshi,[†] Kazue Tsuchimori,[†] Kenichi Hamagami,[†] Masanori Watanabe, [*,‡] Masako Sasaki,[†] Youko Ishihara,[§] Kimio Tohyama,[†] Tomoyuki Kitazaki,[†,||] Tsuyoshi Maekawa,[‡] and Minoru Sasaki[*,†]*

[†] Research, Takeda Pharmaceutical Company Ltd., 26-1, Muraokahigashi 2-chome, Fujisawa, Kanagawa 251-8555, Japan.

[‡] Research Division, SCOHIA PHARMA, Inc., 26-1, Muraokahigashi 2-chome, Fujisawa, Kanagawa 251-8555, Japan.

[§] Pharmaceutical Sciences, Takeda Pharmaceutical Company Ltd., 26-1, Muraokahigashi 2-chome, Fujisawa, Kanagawa 251-8555, Japan.

Table of contents

Analytical HPLC traces S2

Analytical HPLC traces

Compound **2a**

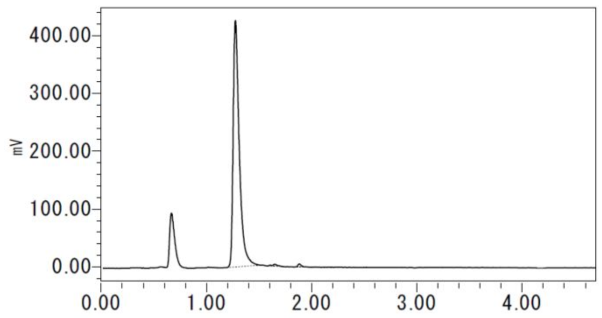

Result of Analysis

	Retention Time (min)	Peak area (μV×sec)	Divisional method	% of Area
1	1.276	1721319	BB	98.75
2	1.649	11292	bb	0.65
3	1.882	10555	BB	0.61

Compound **4a**

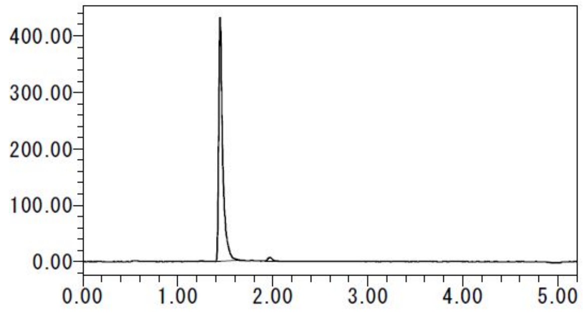

Result of Analysis

	Retention Time (min)	Peak area (μV×sec)	Divisional method	% of Area
1	1.244	1652	bb	0.13
2	1.444	1277510	BB	98.28
3	1.971	20674	BB	1.59

Compound **4b**

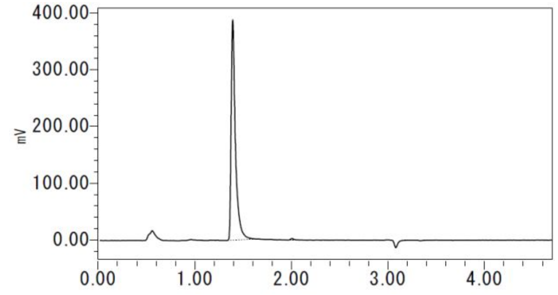

Result of Analysis

	Retention Time (min)	Peak area (μV×sec)	Divisional method	% of Area
1	1.393	1190934	BB	99.62
2	2.005	4588	bb	0.38

Compound **4c**

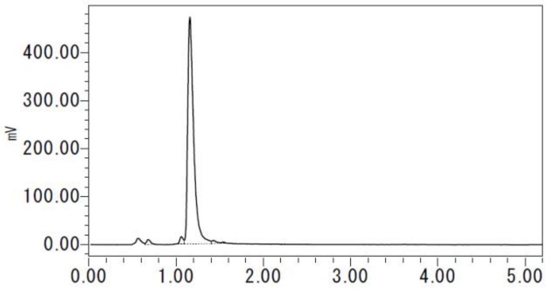

Result of Analysis

	Retention Time (min)	Peak area (μV×sec)	Divisional method	% of Area
1	0.679	34324	VB	1.46
2	1.061	41803	BV	1.78
3	1.158	2243275	VV	95.41
4	1.430	23989	VV	1.02
5	1.541	7885	VB	0.34

Compound **(*R*)-5b**

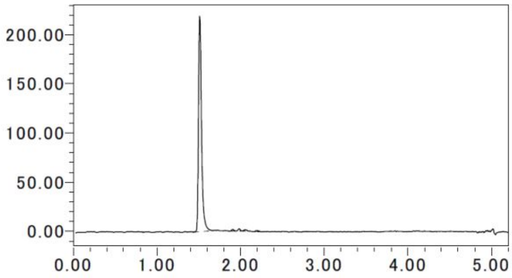

Result of Analysis

	Retention Time (min)	Peak area (μV×sec)	Divisional method	% of Area
1	1.512	554056	BB	97.08
2	1.983	13460	bb	2.36
3	2.197	3234	bb	0.57

Compound **(*S*)-5b**

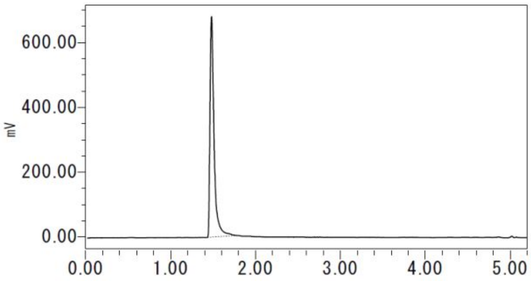

Result of Analysis

	Retention Time (min)	Peak area (μV×sec)	Divisional method	% of Area
1	1.482	2284373	BB	100.00

Compound **6b**

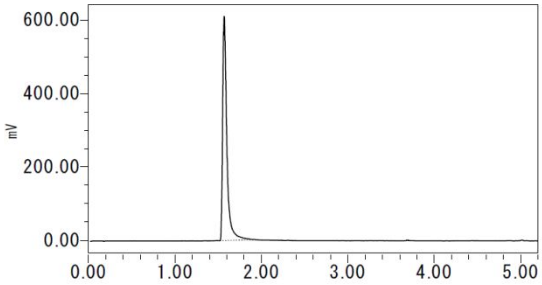

Result of Analysis

	Retention Time (min)	Peak area (μV×sec)	Divisional method	% of Area
1	1.570	2065064	BB	99.69
2	3.693	6378	bb	0.31

Compound **6c**

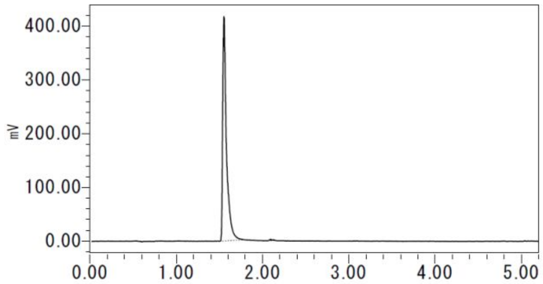

Result of Analysis

	Retention Time (min)	Peak area (μV×sec)	Divisional method	% of Area
1	1.553	1274597	BB	99.33
2	2.092	8638	bb	0.67
